# Antigen-driven colonic inflammation is associated with development of dysplasia in primary sclerosing cholangitis

**DOI:** 10.1038/s41591-023-02372-x

**Published:** 2023-06-15

**Authors:** Dustin G. Shaw, Raúl Aguirre-Gamboa, Marcos C. Vieira, Saideep Gona, Nicholas DiNardi, Anni Wang, Anne Dumaine, Jody Gelderloos-Arends, Zachary M. Earley, Katherine R. Meckel, Cezary Ciszewski, Anabella Castillo, Kelly Monroe, Joana Torres, Shailja C. Shah, Jean-Frédéric Colombel, Steven Itzkowitz, Rodney Newberry, Russell D. Cohen, David T. Rubin, Christopher Quince, Sarah Cobey, Iris H. Jonkers, Christopher R. Weber, Joel Pekow, Patrick C. Wilson, Luis B. Barreiro, Bana Jabri

**Affiliations:** 1 Committee on Immunology, University of Chicago, Chicago, IL USA; 2 Department of Medicine, University of Chicago, Chicago, IL USA; 3 Committee on Genetics, Genomics and Systems Biology, University of Chicago, Chicago, IL USA; 4 Department of Ecology and Evolution, University of Chicago, Chicago, IL USA; 5 Department of Genetics, University of Groningen, University Medical Center Groningen, Groningen, the Netherlands; 6 Division of Gastroenterology, Icahn School of Medicine at Mount Sinai, New York, NY USA; 7 Department of Internal Medicine, Washington University School of Medicine, St. Louis, MO USA; 8 Division of Gastroenterology, Hospital Beatriz Ângelo, Loures, Portugal; 9 Division of Gastroenterology, Hospital Luz, Lisboa, Portugal; 10 Faculty of Medicine, Universidade de Lisboa, Lisboa, Portugal; 11 Division of Gastroenterology, University of California San Diego, San Diego, CA USA; 12 Jennifer Moreno VA San Diego Healthcare System, San Diego, CA USA; 13 University of Chicago Inflammatory Bowel Disease Center, Chicago, IL USA; 14 Organisms and Ecosystems, Earlham Institute, Norwich, NR4 7UZ UK; 15 Warwick Medical School, University of Warwick, Coventry, CV4 7HL UK; 16 Gut Microbes and Health, Quadram Institute, Norwich, NR4 7UQ UK; 17 Department of Pathology, University of Chicago, Chicago, IL USA; 18 Section of Rheumatology, University of Chicago, Chicago, IL USA; 19 Department of Pediatrics, University of Chicago, Chicago, IL USA

**Keywords:** Cellular immunity, Chronic inflammation, Inflammatory bowel disease

## Abstract

Primary sclerosing cholangitis (PSC) is an immune-mediated disease of the bile ducts that co-occurs with inflammatory bowel disease (IBD) in almost 90% of cases. Colorectal cancer is a major complication of patients with PSC and IBD, and these patients are at a much greater risk compared to patients with IBD without concomitant PSC. Combining flow cytometry, bulk and single-cell transcriptomics, and T and B cell receptor repertoire analysis of right colon tissue from 65 patients with PSC, 108 patients with IBD and 48 healthy individuals we identified a unique adaptive inflammatory transcriptional signature associated with greater risk and shorter time to dysplasia in patients with PSC. This inflammatory signature is characterized by antigen-driven interleukin-17A (IL-17A)^+^ forkhead box P3 (FOXP3)^+^ CD4 T cells that express a pathogenic IL-17 signature, as well as an expansion of IgG-secreting plasma cells. These results suggest that the mechanisms that drive the emergence of dysplasia in PSC and IBD are distinct and provide molecular insights that could guide prevention of colorectal cancer in individuals with PSC.

## Abstract (web summary)

Multiomics analysis of right colon tissues identifies a distinct immune gene signature associated with greater risk of and shorter time to dysplasia in patients with primary sclerosing cholangitis, but not in patients with inflammatory bowel disease.

## Main

Primary sclerosing cholangitis (PSC) is a chronic immune-mediated liver disease with a strong human leukocyte antigen (HLA) association^1^, which is characterized by liver fibrosis^2^ and is often concomitant with inflammatory bowel disease (IBD)^3^. Colorectal neoplasia (CRN) is a major complication of patients with PSC and IBD^4^, with a 50% 25-year cumulative risk for CRN, which is five times greater than what is observed in patients with IBD without PSC^5^.

The duration and severity of inflammation in IBD are known to correlate with CRN development^6,7^. Generally, inflammation is thought to impact cancer development at many stages, including initiation (introduction of mutations into proliferating cells) and promotion (preferential expansion of mutated cells via external proliferation signals)^8^. In IBD without PSC, reactive oxygen species (ROS) are thought to introduce DNA mutations in colonic epithelial cells, which then preferentially expand in response to proliferation signals^9,10^. Therefore, IBD inflammation is probably relevant to the initiation of CRN. Whether or not IBD inflammation is associated with the promotion of CRN once mutations have been established is unclear. Furthermore, whether the mechanism for driving CRN in PSC is the same as in IBD has not been investigated. The substantially higher risk of CRN in PSC, the limited genetic overlap between IBD and PSC^11^, and the unique presentation of colitis in PSC^12,13^ suggest collectively that the pathogenesis of CRN in PSC and IBD may be distinct.

**Fig. 1:**
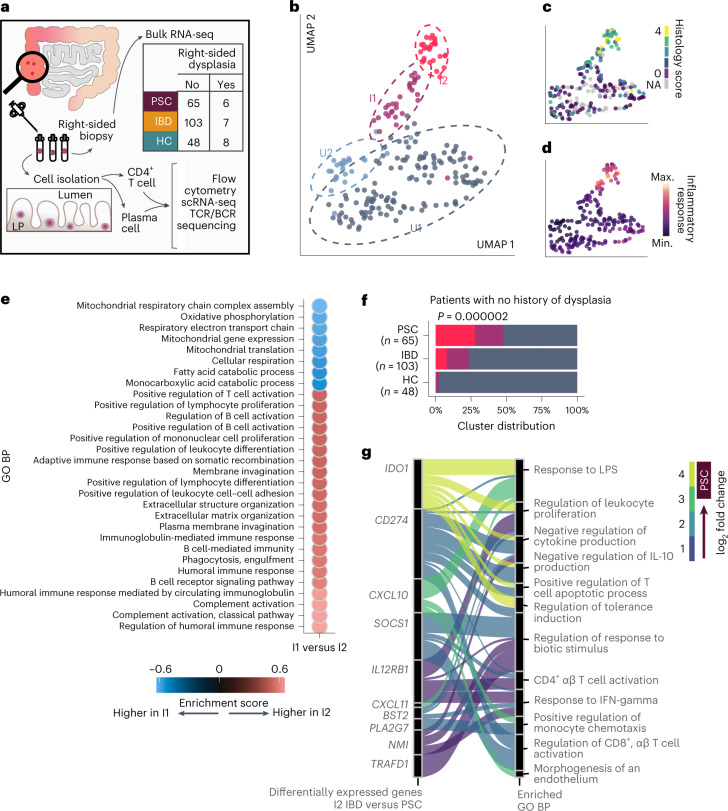
A subset of patients with PSC with no history of dysplasia show a unique and highly inflamed transcriptional profile. **a**, Graphical representation of the methodology of this study. To control for biological differences across the span of the colon, we collected 8–10 tissue biopsies only from the right colon. Our patient cohort included patients with PSC, patients with IBD with a history of right-sided colitis and patients with no history of IBD or PSC (HCs). Patients were retrospectively determined to have right-sided dysplasia. From one biopsy, we isolated RNA for whole-tissue bulk RNA-seq. The remainder of the biopsies were mechanically and enzymatically disrupted to isolate lamina propria CD4^+^ T cells and plasma cells for analysis via flow cytometry, scRNA-seq, and TCR and B cell receptor (BCR) analysis. **b**–**d**, Uniform manifold approximation and projection (UMAP) plots using right colon tissue samples from individuals with no history of dysplasia at the time of sample collection (*n* = 65 with PSC, *n* = 103 with IBD, *n* = 48 HCs). Samples are annotated by transcriptionally determined cluster (**b**), histologically scored inflammation (**c**) or inflammatory response gene set enrichment score (**d**). **e**, Dot plot showing the top 30 most significantly enriched GO biological processes (BPs) (GSEA test *q* < 0.1 × 10^−9^) between the I2 and I1 clusters; the color represents the enrichment score and direction of effect. **f**, Distribution of individuals across clusters among individuals without dysplasia; statistical significance was determined using a chi-squared test. Red corresponds to cluster I2, purple to cluster I1 and blue to cluster U (combined U1 and U2). **g**, Alluvial plot connecting the top enriched GO BPs with the significantly upregulated genes in I2 PSC versus I2 IBD; connections are colored according to fold change. Max., maximum; min., minimum.

To identify the mechanisms that underlie the development of CRN in PSC, we transcriptionally and cellularly profiled 71 patients with PSC (93% of whom had a diagnosis of IBD, collectively referred to as ‘PSC’), 110 patients with IBD without PSC (IBD) and 56 healthy individuals (healthy controls (HCs)), including patients with and without active dysplasia (an early stage of CRN). Our analysis included broad, unbiased tissue transcriptional profiling combined with flow cytometry analysis (Fig. 1a). In addition, given the strong HLA association with PSC but not IBD, we performed single-cell transcriptomics of T cells and plasma cells with T cell receptor (TCR) and immunoglobulin analysis to evaluate the hypothesis that T and B cell antigen-driven responses contribute to the development of CRN in patients with PSC. We focused on the right colon because inflammation and dysplasia are most often right-sided in patients with PSC^14,15^.

Our study found that the nature of inflammation and the mechanisms promoting dysplasia are distinct between PSC and IBD, and that PSC inflammation may be antigen-driven.

## Results

### PSC and IBD show markedly different inflammatory signatures

To characterize differences in the tissue environment of patients with PSC and IBD, we performed RNA sequencing (RNA-seq) on colon tissue from patients who had no history of dysplasia in any segment of the colon (the clinical and demographic data of these patients is summarized in Extended Data Table 1). This included samples from 65 patients with PSC, 103 patients with IBD and 48 HCs with no history of dysplasia. The colon was biopsied at the same location (10 cm distal to the ileocecal valve; right colon) to avoid bias related to regional immune and microbial differences across the colon^16^. We sampled the right colon because nearly all patients with PSC have a history of inflammation in the right colon^14^ and dysplasia is most common in the right colon of individuals with PSC^15^. Although colitis in IBD is not always right-sided^17^, we only enrolled patients with IBD with a documented history of right-sided inflammation.

**Extended Data Fig. 1:**
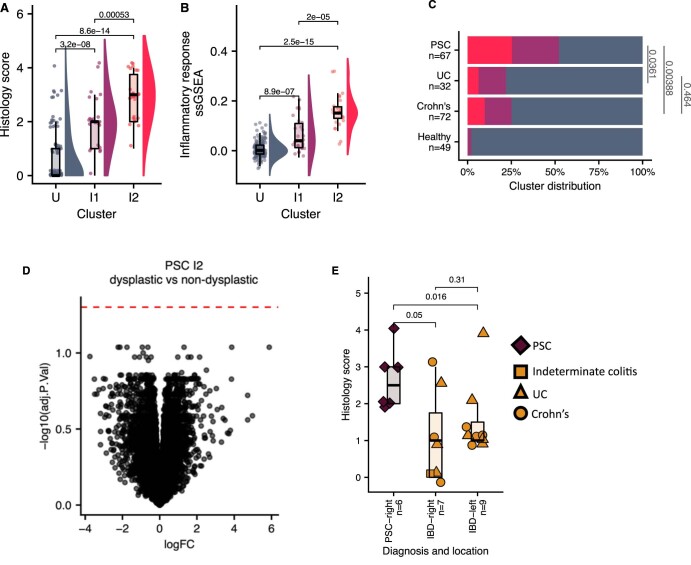
I2 subjects are inflamed. **a**, Histologically-scored inflammation in the right colon of patients without a history of dysplasia, separated by transcriptionally-determined cluster. 0 = no diagnostic abnormality, 1 = quiescent inflammation, 2 = mild inflammation, 3 = moderate inflammation, 4 = severe inflammation. Significance determined by two-sided, unpaired Wilcoxon test without adjustment for multiple comparisons. **b**, Single sample gene set analysis (ssGSEA) score for the Inflammatory Response gene set (HALLMARK_INFLAMMATORY_RESPONSE, Molecular Signatures Database v7.5.1) calculated from the right colon tissue transcriptome of patients without a history of dysplasia, separated by transcriptionally-defined cluster. Significance determined by two-sided, unpaired Wilcoxon test without adjustment for multiple comparisons. n = 34 I2, 26 I1, 156 U (**a**, **b**). **c**, Distribution of subjects with no history of dysplasia across clusters, statistical significance determined by two-sided Fisher t-test. **d**, Volcano plot summarizing the differentially expressed gene analysis of PSC I2 subjects with right-sided dysplasia versus PSC I2 subjects with no history of dysplasia (n = 5 PSC I2 with dysplasia, 17 PSC I2 without dysplasia). 0 genes passed the threshold of significance (red dashed line, adjusted p-value > 0.05), suggesting that the transcriptional profile of PSC I2 subjects is identical whether or not the patient has right-sided dysplasia. **e**, Histologically-scored inflammation in patients with PSC and IBD that have dysplasia, separated by the location of dysplasia (n = 6 PSC-right, 7 IBD right-sided dysplasia, 9 IBD left-sided dysplasia. Significance determined by two-sided, unpaired Wilcoxon test without adjustment for multiple comparisons. Center line represents the median value; hinges indicate the 1st and 3rd quartiles; upper and lower whiskers extend to the largest and smallest values that are within 1.5 times the interquartile range from 1st and 3rd quartiles, respectively (**a**,**b**,**e**).

Unsupervised clustering analysis using the 3,000 most hypervariable genes across diagnoses identified four distinct clusters of patients (Fig. 1b). Two clusters, uninflamed 1 and 2 (U1 and U2), were histologically and transcriptionally uninflamed (Fig. 1c,d and Extended Data Fig. 1a,b) and were therefore combined (collectively referred to as ‘U’) in subsequent analyses. Two clusters of patients with inflammation were identified and labeled inflamed 1 and 2 (I1 and I2), with I2 being more inflamed than I1 (Extended Data Fig. 1a,b). Genes significantly upregulated in I2 compared to I1 (*n* = 7,734, 51% of all genes tested with a false discovery rate (FDR) < 5%) were strongly enriched among gene ontology (GO) terms related to both innate and adaptive immune pathways (Fig. 1e).

The distribution of diagnoses was markedly different across transcriptional clusters (Fig. 1f). Nearly all HCs fell in cluster U, whereas there was an enrichment of patients with IBD, and to a greater extent patients with PSC, in clusters I1 and I2. Cluster I2 had the greatest difference in proportion of PSC and IBD: 27% of patients with PSC versus 7% among patients with IBD (chi-squared *P* = 2.0 × 10^−6^). This difference persisted when comparing PSC separately to either Crohn’s disease (chi-squared *P* = 0.003) or ulcerative colitis (chi-square *P* = 0.01; Extended Data Fig. 1c). There was no difference between Crohn’s disease and ulcerative colitis in the distribution of patients across transcriptional clusters (chi-squared *P* = 0.84). Therefore, we compared PSC to IBD without distinction of Crohn’s disease or ulcerative colitis in all subsequent analyses. Because patients with IBD or PSC can have the I2 signature (albeit at different frequencies), we investigated whether there were any features unique to PSC I2 compared to IBD I2. We observed immune pathways enriched in PSC I2 (Fig. 1g), including pathways related to T cell activation and response to bacterial molecules. Therefore, although both PSC and IBD I2 are inflamed, the nature of these inflammations was transcriptionally distinct. Of note, some of the genes belonging to the pathways enriched in PSC I2 were previously associated with PSC using genome-wide association studies (Fig. 1g; for example, IDO1 and SOCS1)^18^.

These findings provide credence to the long-standing hypothesis that the nature of PSC and IBD inflammation is different^12^—a hypothesis based on clinical observations of distinct patterns of inflammation in PSC. The features unique to PSC inflammation might also provide clues into potential mechanisms of dysplasia in PSC.

### PSC dysplasia has a unique inflammatory signature

Next, we investigated whether the I2 PSC signature was related to the development of dysplasia. To do so, we performed RNA-seq analysis on nondysplastic mucosa from patients with right-sided dysplasia detected at the time of sampling (the clinical and demographic data of these patients are summarized in Extended Data Table 2). This included six patients with PSC and dysplasia, seven patients with IBD and dysplasia, and eight control patients with dysplasia (sporadic dysplasia). Because we did not specifically sample dysplastic tissue, we analyzed the tissue environment in which dysplasia developed rather than the dysplastic lesion itself.

**Fig. 2:**
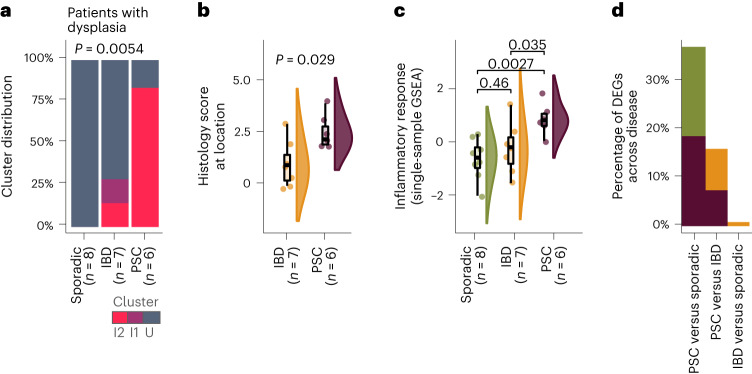
The colonic dysplasia landscape of PSC is enriched in the I2 signature and differs from that of IBD. **a**–**d**, Patients with active right-sided dysplasia were analyzed (*n* = 6 with PSC, *n* = 7 with IBD, *n* = 8 sporadic). **a**, Distribution of individuals with right colon dysplasia across clusters; statistical significance was determined using a chi-squared test. **b**, Histologically scored inflammation at the site of dysplasia within the right colon using the UCM histological criteria for grading disease activity: 0, no diagnostic abnormality; 1, quiescent or minimally active; 2, mild; 3, moderate; and 4, severe. The right colon includes the cecum, ascending colon and hepatic flexure. Significance was determined using a double-sided Wilcoxon rank-sum test. **c**, Single-sample gene set enrichment analysis (GSEA) inflammatory response score calculated from the transcriptome of the right colon tissue biopsy. Significance was determined using a double-sided Wilcoxon rank-sum test without adjustment for multiple comparisons. The center line represents the median; the hinges indicate the first and third quartiles; the upper and lower whiskers extend to the largest and smallest values that are within 1.5 times the interquartile range (IQR) from the first and third quartiles, respectively (**b**,**c**). **d**, Bar graph quantifying the percentage of differentially expressed genes (DEGs) (adjusted *P* < 0.05) in each comparison (the proportion of genes upregulated in PSC are shown in purple, genes upregulated in sporadic dysplasia are shown in green, and genes upregulated in IBD are shown in orange).

Using the cluster signatures generated from patients with no history of dysplasia, we built a classification model using a regularized logistic regression (elastic net (eNet); Methods) to predict the cluster assignment of patients with right-sided dysplasia. Validation showed that our prediction model had perfect accuracy in ascribing cluster I2 (area under the curve (AUC) = 1, *n* = 53). Strikingly, 83% of patients with PSC and right-sided dysplasia were assigned to cluster I2. In contrast, 0% of control patients with right-sided sporadic dysplasia and 14% of patients with IBD and right-sided dysplasia were classified as I2 (Fig. 2a). Importantly, among patients with PSC classified as I2 we found no differences in gene expression between patients with and without right-sided dysplasia (Extended Data Fig. 1d), suggesting that the I2 PSC signature is not impacted by the presence of dysplasia and may reflect an immunological and transcriptional state promoting the development of dysplasia.

Consistent with the strong overlap between PSC dysplasia and the I2 transcriptional signature, we observed higher inflammation levels, both histologically (Fig. 2b) and transcriptionally (Fig. 2c), in the tissue environment where PSC dysplasia developed versus those environments of IBD dysplasia or sporadic dysplasia. Additionally, we observed greater histologically scored inflammation in right-sided PSC dysplasia compared to left-sided IBD dysplasia; there was no difference in inflammation between left-sided and right-sided IBD dysplasia (Extended Data Fig. 1e). This suggests that our results are not due to sampling from the right colon of patients with IBD.

We then tested for transcriptional differences across diagnosis and surprisingly found no genes differentially expressed between IBD dysplasia and sporadic dysplasia-associated tissue (Fig. 2d). This is consistent with previous studies showing no differences in the proinflammatory molecular subtype between IBD and sporadic colorectal cancers (CRCs)^9,19^. In contrast, 15% and 36% of all genes tested (*n* = 15,146) were differentially expressed when contrasting PSC dysplasia with IBD dysplasia and sporadic dysplasia, respectively (Fig. 2d).

Taken together, this suggest that inflammation plays a different role in the development of CRN in IBD versus PSC. In IBD, because the tissue environment at the time of dysplasia is uninflamed and transcriptionally indistinguishable from sporadic dysplasia, we propose that while inflammation contributes to the initiation of CRN^20^, it may not have an important role in the promotion of CRN. In contrast, PSC dysplasia is nearly always found in an inflamed environment, suggesting that inflammation may play a role in the oncogenic progression of PSC. Whether or not inflammation contributes to the initiation of CRN in PSC remains to be determined.

### Evidence for antigen-driven immune responses in PSC

**Fig. 3:**
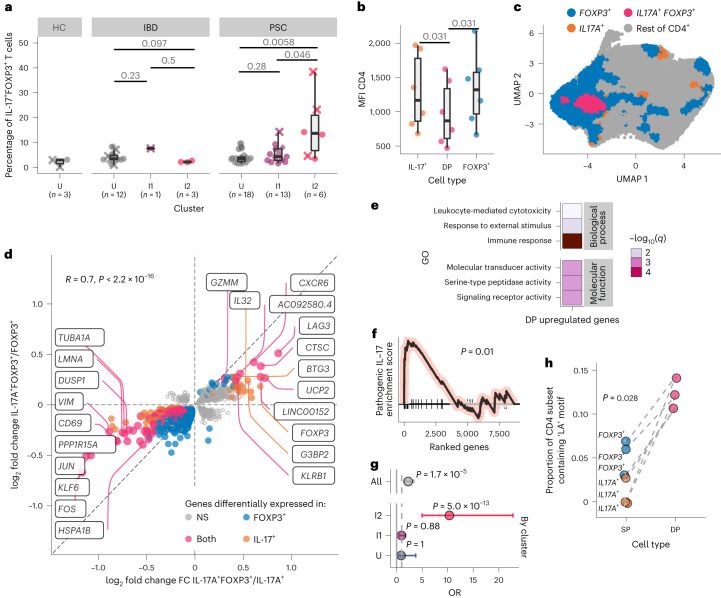
PSC inflammation is characterized by IL-17A^+^FOXP^+^ CD4 T cells enriched for TCRs containing LA. **a**, Percentage of right colon lamina propria CD4 T cells positive for IL-17A and FOXP3 (*n* = 6 PSC I2, *n* = 13 PSC I1, *n* = 18 PSC U, *n* = 3 IBD I2, *n* = 1 IBD I2, *n* = 12 IBD U, *n* = 3 NC U). Significance was determined using a double-sided Wilcoxon rank-sum test without adjustment for multiple comparisons. The ‘x’ denotes patients with dysplasia at the time of sampling. **b**, Mean fluorescence intensity (MFI) of surface CD4 expression of cells from patients in the I2 PSC group (*n* = 6). Significance was determined using a double-sided Wilcoxon matched-pairs signed-rank test without adjustment for multiple comparisons (*n* = 6). The center line represents the median; the hinges indicate the first and third quartiles; the upper and lower whiskers extend to the largest and smallest values that are within 1.5 times the IQR from the first and third quartiles, respectively (**a**, **b**). **c**, UMAP of CD4 T cells from patients with PSC, annotated according to cell type (*n* = 15 patients, *n* = 25,942 cells). **d**, log_2_ fold change of gene expression comparing DP to *FOXP3*^+^ CD4 T cells (*x* axis) or *IL17A*^+^ CD4 T cells (*y* axis) among I2 PSC individuals (*n* = 4 patients with PSC I2). Each gene is a circle colored if significantly (*P* < 0.1) changed across a comparison. NS, not significant. The highest fold change genes are labeled on the graph. **e**, Most significantly enriched gene sets upregulated in DP CD4 T cells versus either *IL17A*^+^ or *FOXP3*^+^ CD4 (*n* = 4 patients with PSC I2). **f**, Enrichment plot for a pathogenic IL-17 signature based on ranking the genes on their changes in expression in DP CD4 versus *IL17A*^+^ CD4 cells. The GSEA nominal *P* value is shown (*n* = 4 patients with PSC I2). **g**, Top and bottom 10% of DP cells by enrichment for pathogenic IL-17 signature were identified and ORs of top vs bottom 10% by cluster are presented as a Forest plot with the 95% confidence intervals and labeled *P* value (Fisher exact test). (I2, I1 or U, *n* = 15 PSC patients, *n* = 25,942 cells). **h**, Proportion of I2 PSC TCRβ chains containing the LA motif (*n* = 4). The dashes denote values from the same patient. Significance was determined using a double-sided, unpaired Wilcoxon rank-sum test.

**Extended Data Fig. 2:**
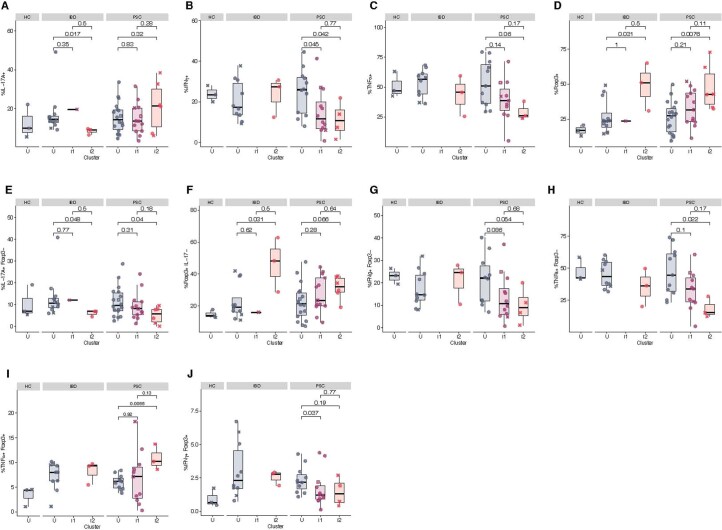
Cytokines secreted by CD4 T cells across transcriptional clusters. **a–d**, Proportion of right colon lamina propria CD4 T cells expressing IL-17A (**a**), IFNγ (**b**), TNFα (**c**), or FOXP3 (**d**) after 3 hours of stimulation with PMA/ionomycin. **e**, Proportion of right colon lamina propria CD4 T cells that are IL-17A^+^ FOXP3^negative^ after 3 hours of stimulation with PMA/ionomycin. **f**, Proportion of right colon lamina propria CD4 T cells that are FOXP3^+^ IL-17A^negative^ after 3 hours of stimulation with PMA/ionomycin. **g**, **h**, Proportion of right colon lamina propria cells that are FOXP3^negative^ and IFNγ^+^ (**g**) or TNFα^+^ (**h**) after 3 hours of stimulation with PMA/ionomycin. **i**, **j**, Proportion of right colon lamina propria cells that are FOXP3^+^ and IFNγ^+^ (**i**) or TNFα^+^ (**j**) after 3 hours of stimulation with PMA/ionomycin. **a–j**, Significance determined by two-sided, unpaired Wilcox test without adjustment for multiple comparisons. A total of 6 PSC I2, 13 PSC I1, 18 PSC U, 3 IBD I2, 1 IBD I1, 12 IBD U, and 3 HC were included in the intracellular flow cytometry analysis. Not all samples were stained with every marker, resulting in a lower number than the total samples being included in the plots above. Center line represents the median value; hinges indicate the 1st and 3rd quartiles; upper and lower whiskers extend to the largest and smallest values that are within 1.5 times the interquartile range from 1st and 3rd quartiles, respectively.

Given the enrichment of CD4 T cell activation in PSC I2 (Fig. 1g), we measured the expression of canonical markers associated with activation (interleukin-17A (IL-17A), interferon-γ (IFNγ), tumor necrosis factor-α (TNFα)) and regulation (forkhead box protein P3 (FOXP3)) on lamina propria CD4 T cells. Although we did not see increases in the expression of any single marker (Extended Data Fig. 2a–d), we found an increase in IL-17A^+^FOXP3^+^ double-positive (DP) CD4 T cells in patients with PSC classified as I2, relative to patients with PSC classified as U (Fig. 3a) or patients with IBD classified as I2 (*P* = 0.024). These results were particularly interesting given the previous implication of IL-17A^+^FOXP3^+^ CD4 T cells in the development of CRN^21^. The DP T cells in PSC I2 had lower surface expression of CD4 than their IL-17A^+^ and FOXP3^+^ single-positive (SP) counterparts (Fig. 3b), suggesting that DP cells were more activated or chronically stimulated^22^. There was no increase in IL-17A^+^FOXP3^−^, FOXP3^+^IL-17^−^, IFNγ^+^FOXP3^−^ or TNFα^+^FOXP3^−^ CD4 T cells, nor an increase in IFNγ^+^FOXP3^+^ or TNFα^+^FOXP3^+^ DP cells in PSC I2 compared to IBD I2 (Extended Data Fig. 2e–j).

**Extended Data Fig. 3:**
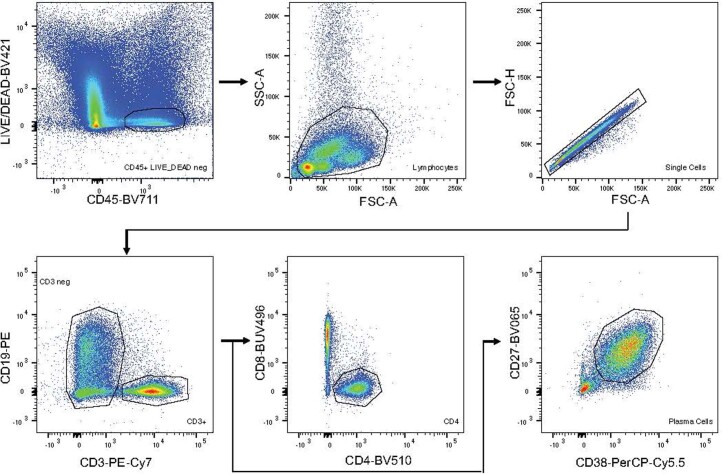
Gating strategy used for sorting of CD4 T cells and plasma cells. CD45^+^ live cells were gated for lymphocytes, and then singlets. CD4 T cells were sorted from this singlet population as CD3^+^ CD19 negative, CD4^+^ CD8 negative. Plasma cells were sorted from this singlet population as CD3 negative, CD27^+^ CD38^+^.

**Extended Data Fig. 4:**
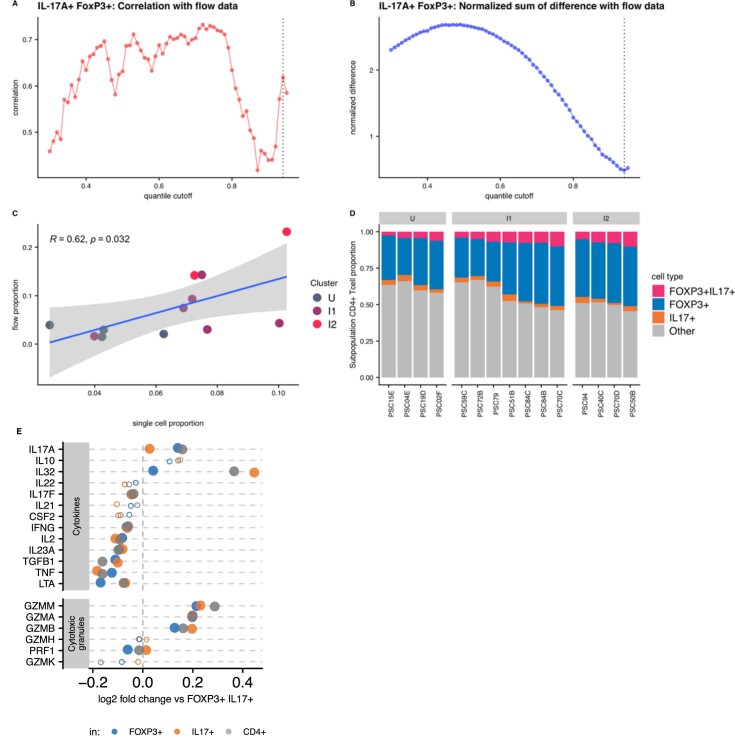
Transcriptional identification of the IL17-A^+^ FOXP3^+^ CD4 T cells. **a**, Correlation of proportion of IL-17A^+^ FOXP3^+^ cells by flow cytometry versus scRNAseq at each quantile cutoff value used to identify positive (*IL17A*+ *FOXP3*^+^) cells. **b**, Normalized sum of differences in proportions between proportion of IL-17A^+^ FOXP3^+^ cells by flow cytometry and scRNAseq at each quantile cutoff value used to identify positive (*IL17A*+ *FOXP3*^+^) cells. **c**, Correlation of proportion of IL-17A^+^ FOXP3^+^ cells by flow cytometry versus scRNAseq at the quantile cutoff value used in Fig. 3 (0.94). Significance and correlation determined by two-sided Pearson correlation test. **d**, Proportion of each transcriptionally-determined cell type within total CD4 cells by patient (n = 4 PSC I2, 7 PSC I1, 4 PSC U). **A-C**, n = 2 PSC I2, 6 PSC I1, 4 PSC U. **D**, Log 2 fold change of cytokine expression of double positive cells as compared to *IL17A* single positive (orange), *FOXP3* single positive (blue), or *IL17A* *FOXP3* double negative (gray) (n = 4 PSC I2). Filled circles represent genes significantly changed at adjusted p < 0.1, and open circles represent genes that are not significantly changed adjusted p < 0.1.

We hypothesize that DP CD4 T cells probably have a key role in promoting the unique dysplastic program seen in patients with PSC. To address this hypothesis, we assessed the transcriptional program of DP CD4 T cells using single-cell RNA-seq (scRNA-seq) on freshly isolated right colon lamina propria CD4 T cells (gating strategy exemplified in Extended Data Fig. 3) from patients with PSC (*n* = 5 I2, *n* = 6 I1 and *n* = 4 U). By calibrating the threshold of transcriptional detection of cells coexpressing *IL17A* and *FOXP3* transcripts using our flow cytometry data (Extended Data Fig. 4a–d), we identified both DP and SP cells by scRNA-seq (Fig. 3c). We performed differential expression analysis between the DP and each of the SP populations. This analysis demonstrated that *IL17A*^+^*FOXP3*^+^ DP CD4 T cells were transcriptionally distinct from either *IL17A*^+^ or *FOXP3*^+^ CD4 SP cells (Fig. 3d). Of note, the *GZMM*^23^ and *IL32* (ref. ^24^) genes previously implicated in the development of dysplasia, were both significantly increased in *IL17A*^+^ *FOXP3*^+^ DP CD4 T cells compared to either *FOXP3*^+^ or *IL17A*^+^ SP cells (adjusted *P* < 0.1; Extended Data Fig. 4e). Furthermore, there was an enrichment of GO pathways related to the response to external stimuli, molecular transducer activity and signaling receptor activity in *IL17A*^+^*FOXP3*^+^ DP CD4 T cells compared to both *FOXP3*^+^ and *IL17A*^+^ SP cells (Fig. 3e), which is consistent with the downregulation of CD4 (Fig. 3b) and supports the notion of DP CD4 T cells being chronically activated. Moreover, there was an enrichment for a pathogenic IL-17 signature^25^ in *IL17A*^+^*FOXP3*^+^ DP CD4 T cells (Fig. 3f). We generated an IL-17 pathogenic signature score for all CD4 cells and found that the top 10% of cells were significantly enriched in *IL17A*^+^*FOXP3*^+^ DP CD4 T cells (odds ratio (OR) = 2.27, *P* = 1.65 × 10^−5^, Fisher exact test) (Fig. 3g). Furthermore, once the same test was performed taking into account patient cluster classification (that is, I1, I2 or U), this enrichment was found in I2 patients (OR = 10.3, *P* = 4.96 × 10^−13^, Fisher exact test), although neither in U (OR = 0.89, *P* = 1) nor I1 patients (OR = 0.94, *P* = 0.8) (Fig. 3g). Collectively, these results suggest a pathogenic role for *IL17A*^+^*FOXP3*^+^ DP CD4 T cells in the promotion of CRN in PSC, perhaps via secretion of IL-17A in conjunction with other pro-oncogenic factors such as IL-32 (ref. ^24^) and GZMM^23^ (Extended Data Fig. 4e).

**Extended Data Fig. 5:**
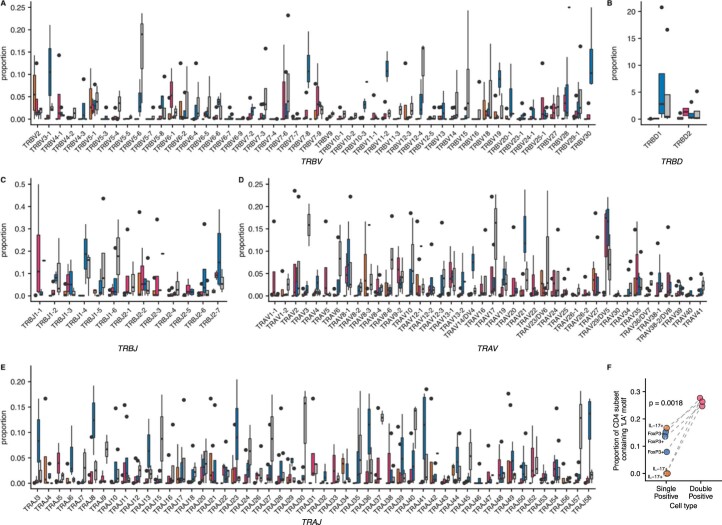
V(D)J usage by cell type in I2 PSC. **a-e**, *TRBV* (**a**), *TRBD* (**b**), *TRBJ* (**c**), *TRAV* (**d**) and *TRAJ* (**e**) gene usage by cell type amongst CD4 T cells from I2 PSC patients. **f**, Proportion of cells containing amino acid motif ‘LA’ in the TCR beta chain by cell type amongst I2 PSC patients using TRBD2. Gray lines denote paired values from the same patients. SP = ‘single positive’, DP = ‘double positive’ i.e. *IL17A*^+^ *FOXP3*^+^. Significance determined by two-sided, unpaired Wicoxon text. **a-f**, Datapoints and box plot color denotes cell type (pink = *IL17A*^+^ *FOXP3*^+^ DP, orange = *IL17A*+ SP, blue = *FOXP3*^+^ SP, gray = negative for *IL17A* and *FOXP3*). n = 3 PSC I2, 1,858 cells. **a**–**e**, Center line represents the median value; hinges indicate the 1st and 3rd quartiles; upper and lower whiskers extend to the largest and smallest values that are within 1.5 times the interquartile range from 1st and 3rd quartiles, respectively. Significance test using two-sided, unpaired Wilcoxon test without adjustment for multiple comparisons. No test reached significance (p < 0.05).

**Extended Data Fig. 6:**
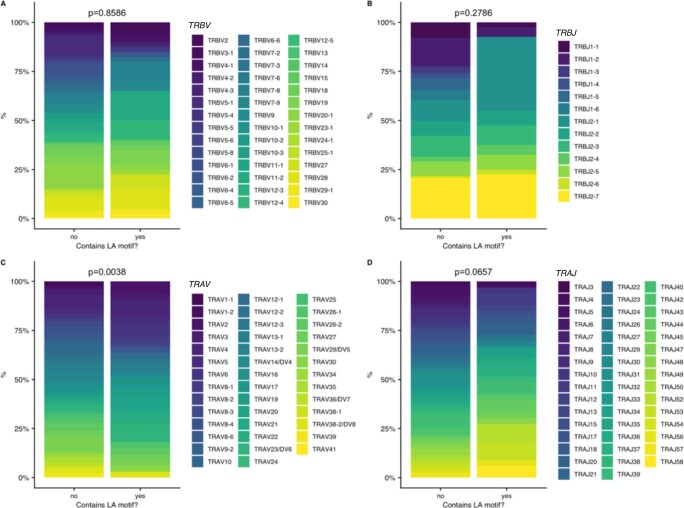
V(D)J gene usage amongst *IL17A*^+^*FOXP3*^+^ CD4 T cells containing ‘LA’ motif. **a–d**, *TRBV* (**a**), *TRBJ* (**b**), *TRAV* (**c**), and *TRAJ* (**d**) gene usage amongst *IL17A*^+^ *FOXP3*^+^ CD4 T cells stratified by whether the Beta chain contains the ‘LA’ amino acid motif. Significance determined by Chi-squared test. n = 3 PSC I2, 1,858 cells.

Finally, to assess whether we could identify signs of an antigen-driven response in the DP CD4 T cells, we searched for a TCR motif enriched in the non-germline-encoded, complementarity-determining region 3 (CDR3) of the *IL17A*^+^*FOXP3*^+^ DP T cell subset. While we did not find any preferential V, D or J gene use in either the TCRβ or TCRα chains (Extended Data Fig. 5a–e), we identified an enrichment for the ‘leucine-alanine (LA)’ amino acid motif (Fig. 3h). LA is a germline-encoded motif that exists in only one of the possible open reading frames (ORFs) of TRBD2. Thus, the use of this motif and the ORF-specific use suggest antigen-driven selection of the TCR in the DP CD4 T cell subset. Additionally, a comparison of SP and DP cells using TRBD2 demonstrated a specific enrichment of the LA amino acid motif in DP T cells (Extended Data Fig. 5f), suggesting a preferential selection for this ORF among DP cells. Finally, we analyzed the V and J use among cells containing the LA motif (Extended Data Fig. 6a–d) and found that the Vα gene use of DP cells containing the LA motif were distinct from DP cells without the LA motif (Extended Data Fig. 6c), further suggesting that these DP LA-containing cells have a distinct TCR.

Strong genetic HLA class II association in complex immune disorders implies a pathogenic role for antigen-specific T and B cell responses^18^. In contrast to IBD, PSC is associated with HLA class II (ref. ^1^). PSC is specifically associated with the ancestral AH8.1 (HLA-A*01:01-C*07:01-B*08:01-DRB3*01:01-DRB1*03:01-DQA1*05:01-DQB1*02:01 haplotype) and HLA-DRB1*13:01-DQA1*01:03-DQB1*06:03 haplotypes^26^. The AH8.1 haplotype was observed in all patients with PSC who showed LA-containing DP cell expansions (Extended Data Table 3), which is consistent with the hypothesis that an antigen presented by an HLA class II molecule encoded by this haplotype drives the expansion of LA-containing DP CD4 T cells.

**Fig. 4:**
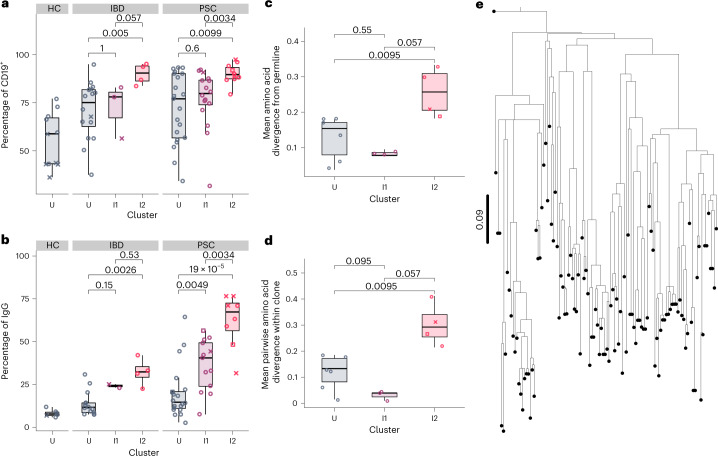
PSC inflammation is characterized by an influx of IgG plasma cells and plasma cells show signs consistent with an antigen drive. **a**, Proportion of right colon plasma cells positive for surface CD19 by flow cytometry (*n* = 10 PSC I2, *n* = 16 PSC I1, *n* = 21 PSC U, *n* = 4 IBD I2, *n* = 3 IBD I1, *n* = 16 IBD U, *n* = 11 HC U). **b**, Proportion of IgG-secreting plasma cells among total right colon plasma cells as determined by enzyme-linked immunosorbent spot (ELISpot) (*n* = 8 PSC I2, *n* = 13 PSC I1, *n* = 20 PSC U, *n* = 4 IBD I2, *n* = 2 IBD I1, *n* = 14 IBD U, *n* = 6 HC U). **c**, Mean amino acid divergence from the inferred germline within CDR3 of the largest clones identified in each patient (*n* = 4 PSC I2, *n* = 3 PSC I1, *n* = 6 PSC U). **d**, Mean pairwise amino acid divergence within CDR3 of the largest clones identified in each patient (*n* = 4 PSC I2, *n* = 3 PSC I1, *n* = 6 PSC U). Each symbol represents an individual patient (open circles denote patients without dysplasia at the time of sampling; ‘x’ denotes patients with dysplasia at the time of sampling; open squares denote patients indefinite for dysplasia at the time of sampling). The center line represents the median; the hinges indicate the first and third quartiles; the upper and lower whiskers extend to the largest and smallest values within 1.5 times the IQR from the first and third quartiles, respectively (**a**–**d**). **e**, Dendrogram of heavy chain sequences within the top clone of an I2 patient (*n* = 110 sequences). This clone demonstrates a ‘lop-sided’ branching pattern, which is consistent with nonrandom mutation accumulation and antigen drive. The origin point represents the inferred germline sequence. The scale bar represents the codon substitutions per codon.

**Extended Data Fig. 7:**
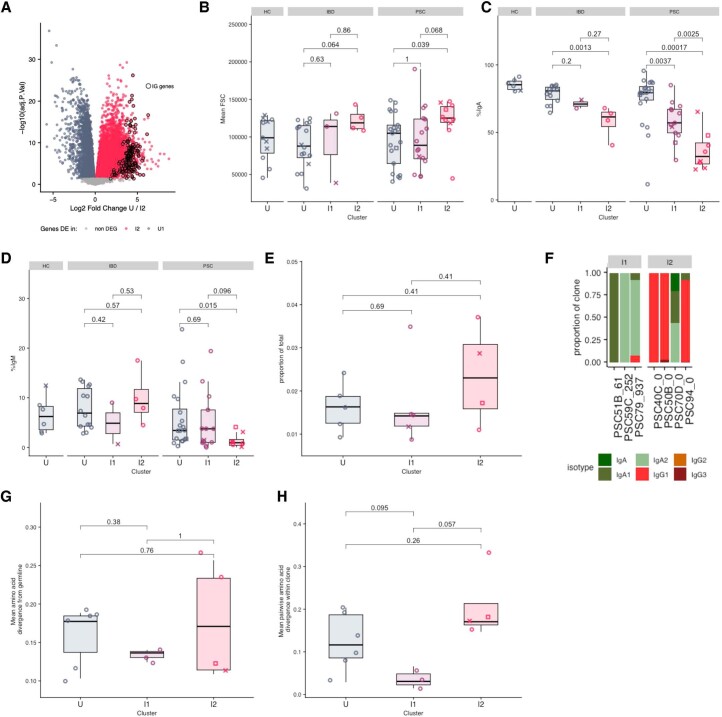
Features of the top plasma cell clones in PSC patients. **a**, Volcano plot of the negative log base 10 adjusted p-value versus log base 2 fold change of the genes differentially expressed in the whole tissue biopsies of I2 versus U patients (n = 34 I2, 133 U). Closed circles denote genes coding for immunoglobulin constant region; heavy chain V, D, or J segments; or light chain V or J segments. **b**, Mean forward scatter (FSC) of right colon plasma cells across clusters as determined by flow cytometry. **c**, Proportion of IgA-secreting plasma cells amongst total right colon plasma cells as determined by ELISpot. **d**, Proportion of IgM-secreting plasma cells amongst total right colon plasma cells as determined by ELISpot. **e**, Proportion of the total repertoire made up by the top clone within each subject. **f**, Proportion of plasma cells of each isotype by clone. **g**, Mean amino acid divergence from inferred germline across entire heavy chain sequence of largest clones identified in each patient. **h**, Mean pairwise amino acid divergence across entire heavy chain sequence of largest clones identified in each patient. (**b–e**, **g–h**) Each symbol represents an individual patient (open circles denote patients without dysplasia at the time of sampling, ‘x’ denote patients with dysplasia at the time of sampling, open squares denote patients indefinite for dysplasia at the time of sampling). Center line represents the median value; hinges indicate the 1st and 3rd quartiles; upper and lower whiskers extend to the largest and smallest values that are within 1.5 times the interquartile range from 1st and 3rd quartiles, respectively. Significance determined by two-sided, unpaired Wilcoxon test without adjustment for multiple comparisons. **b–h**, n = 4 PSC I2, 3 PSC I1, 7 PSC U.

As we found a unique, pathogenic-like T cell population enriched in PSC I2, we probed for a B cell response as well. Tissue RNA-seq showed that immunoglobulin transcripts were among the most strongly upregulated genes in I2 (Extended Data Fig. 7a). Given that plasma cells are the predominant B cell subset of the intestinal lamina propria^27^ and express the highest amount of immunoglobulin, we focused our analysis on plasma cells. We found that PSC I2 plasma cells were nearly 100% surface CD19^+^ (Fig. 4a) and larger than plasma cells in PSC U (Extended Data Fig. 7b), suggesting that these cells are recently arrived, active antibody-secreting cells^28^. We observed an ordinal increase in the proportion of plasma cells secreting IgG across clusters in both IBD and PSC (Fig. 4b). The proportion of plasma cells secreting IgG in PSC I2 was significantly greater than in IBD I2 (*P* = 0.016). A corresponding decrease in the proportion of IgA-secreting and IgM-secreting plasma cells was observed ordinally across clusters (Extended Data Fig. 7c,d). PSC colitis is therefore uniquely characterized by an increased proportion of IgG-secreting plasma cells not seen to the same degree in IBD colitis, even IBD I2.

We performed scRNA-seq on plasma cells derived from patients with PSC across clusters (*n* = 4 I2, *n* = 3 I1 and *n* = 6 U) and determined clonal pools. We analyzed the largest clone from each individual, assuming that the largest clone is the most likely to be chronically activated. The three largest clones were from patients with inflammation (Extended Data Fig. 7e) and were predominantly IgG (specifically IgG1) in I2 and IgA in I1 (IgA1 and IgA2) (Extended Data Fig. 7f). We observed a greater mean amino acid divergence from the inferred germline within the CDR3 of the largest clones of I2 compared to I1 and U (Fig. 4c). This was not the case when we analyzed the entire length of the heavy chain (Extended Data Fig. 7g), meaning that the CDR3 specifically was more heavily mutated and diverse in the top I2 than the top I1 and U clones. The top I2 clones also had higher genetic diversity within the CDR3 than the top clones of I2 and U (Fig. 4d), suggesting that multiple clades within those clones may have acquired affinity-increasing mutations. There was no difference in diversity when analyzing the entire length of the heavy chain (Extended Data Fig. 7h). Finally, a phylogenetic tree of the sequences within the largest clone found in an I2 patient demonstrated lop-sided branching patterns characteristic of selection^29^ (Fig. 4e).

Collectively, these data strongly suggest that the clonal IgG plasma cells in I2 PSC are antigen-driven. The signs of an antigen drive in the plasma cells corroborates the preferential enrichment of a TCR motif among pathogenic DP cells, further suggesting that PSC inflammation and dysplasia are antigen-driven.

### PSC I2 inflammation increases the risk of developing dysplasia

**Fig. 5:**
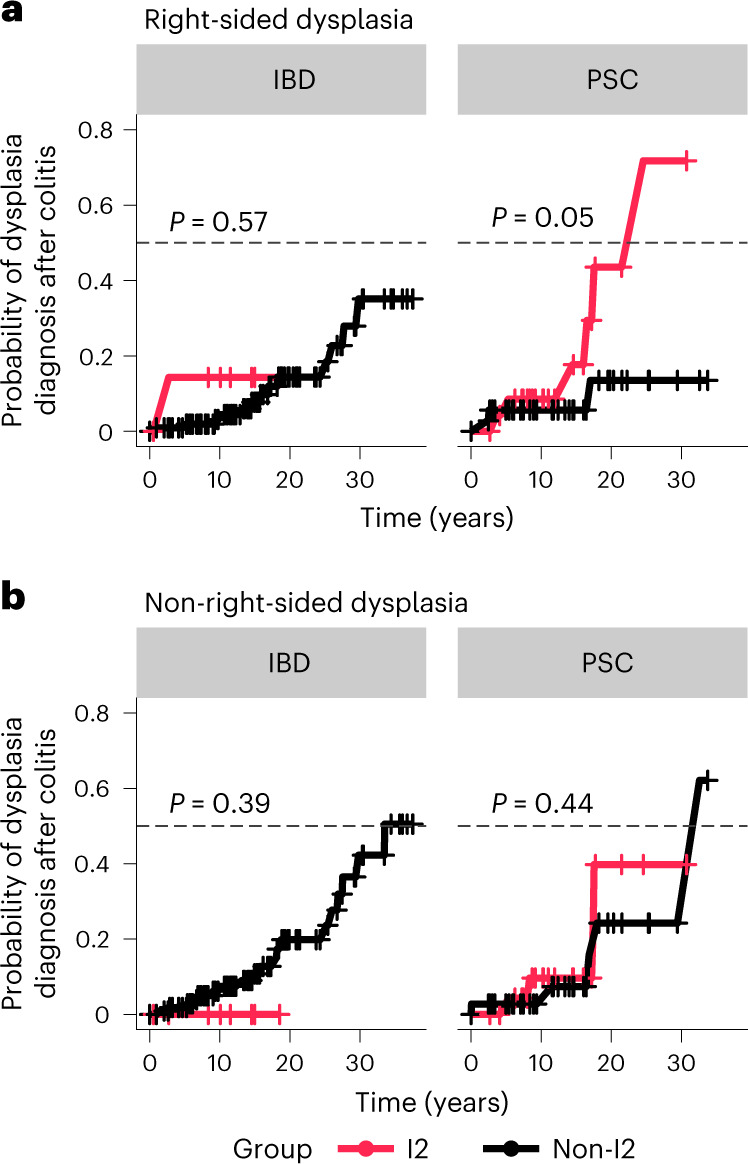
I2 status is associated with a greater risk and shorter time to dysplasia in PSC but not IBD. **a**, Kaplan–Meier-estimated curves for the risk of right-sided dysplasia over time. Patients were classified as I2 (red) or non-I2 (I1 or U, black), according to their transcriptional cluster (*n* = 64 PSC, *n* = 127 IBD). The gray dashed line marks the 0.5 probability of the event (dysplasia diagnosis after colitis). For patients who were sampled at multiple time points, they were classified as I2 if at any point they had an I2 signature, otherwise they were classified as non-I2. Time (years) was calculated from the diagnosis of intestinal colitis to either the first incidence of right-sided colitis or the last colonoscopy recorded. Patients are subset according to diagnosis, that is, IBD (left) or PSC (right). **b**, Kaplan–Meier curves as in **a**, but showing the risk of non-right-sided dysplasia over time (*n* = 64 PSC, *n* = 127 IBD). Statistical outliers for time of follow-up were removed from the analysis before calculating the Kaplan–Meier estimates and *P* value. Individuals are subset according to diagnosis, that is, IBD (left) or PSC (right). Censored points (no dysplasia diagnosed) are marked as +.

If I2 inflammation drives dysplasia in PSC, we expect that patients with PSC classified as I2 will have an increased risk of developing dysplasia compared to patients with PSC who are not I2. To test this, we classified patients with IBD and patients with PSC as I2 or non-I2 (I1 or U). For patients that were sampled at multiple time points, we classified them as I2 if at any point they had an I2 signature; otherwise, they were classified as non-I2. Therefore, we classified patients based on whether they had ever experienced I2 inflammation. We retrospectively calculated the time from the diagnosis of intestinal colitis to either the first incidence of right-sided dysplasia or to the last recorded colonoscopy. Sixty-four patients with PSC were included in this analysis, of which 10 (16%) developed right-sided dysplasia during observation (median 15.5 years). One hundred and twenty-seven patients with IBD were included, of which 17 (13%) developed right-sided dysplasia (median 13.8 years). Of the patients who developed dysplasia, six patients with PSC (60%) and no patients with IBD (0%) were classified as I2. By plotting the Kaplan–Meier-estimated probability of right-sided dysplasia stratified by I2 and non-I2, we found that patients with PSC classified as I2 had a greater risk of developing dysplasia over time than non-I2 patients with PSC (Fig. 5a, right, *P* = 0.05). However, we did not find any difference in the risk of dysplasia between I2 patients with IBD and non-I2 patients with IBD (Fig. 5a, left). This suggests that the I2 signature is associated with a greater risk for right-sided dysplasia in PSC but not IBD. We additionally tested whether right colon I2 status was associated with a greater risk for the development of dysplasia outside the right colon. Of the 64 patients with PSC and 127 patients with IBD in this analysis, 10 patients with PSC (16%) and 23 patients with IBD (18%) developed dysplasia outside the right colon; 5 patients with PSC (50%) and no patients with IBD (0%) of those patients were classified as I2, respectively. We found that I2 was not associated with an increased risk of non-right-sided dysplasia in either PSC or IBD (Fig. 5b), suggesting that I2 inflammation is associated with a greater risk of dysplasia specifically in the region in which it is observed.

## Discussion

The overall goal of our study was to gain insights into the mechanisms driving the high frequency of CRN in PSC and identify a transcriptional signature that could predict development of dysplasia in PSC. A major strength of our study is that we combined tissue RNA-seq with flow cytometry, scRNA-seq, and BCR and TCR repertoire analysis. Furthermore, we controlled for factors such as bacterial load and composition^30^, immune subsets^16^ and epithelial cell function^31^ by restricting our analysis to the right colon. Finally, we included key patient control groups such as patients with and without right-sided dysplasia, and patients with IBD with a history of right-sided inflammation to match the predominant site of inflammation in patients with PSC.

**Fig. 6:**
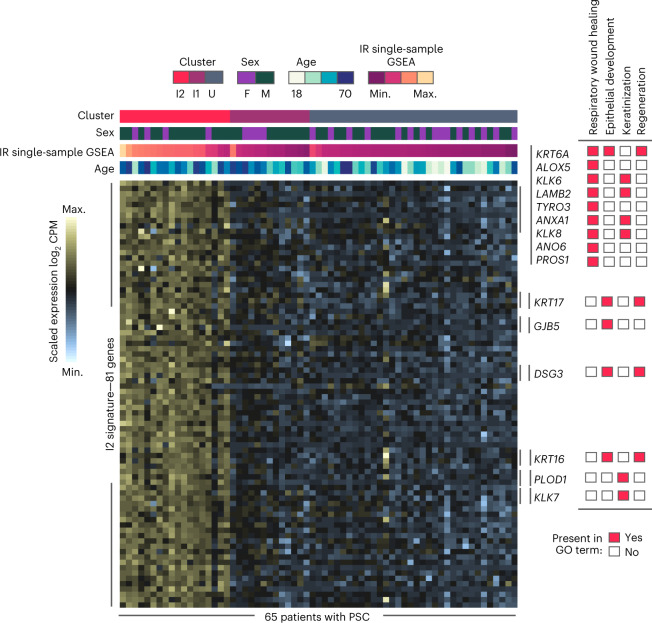
Genes of the I2 PSC classifier model. Heatmap of the expression of the 81 core I2 PSC genes (rows) among PSC patients (columns). Top, the annotation represents the patient characteristics: cluster, sex, age, inflammatory response (IR) and single-sample GSEA score. Annotated on the right are the genes present in wound healing-related GO BPs enriched with the 81 genes from the I2 signature (*n* = 65 patients with PSC).

Collectively, our study reveals that inflammation has a role in the promotion of PSC dysplasia, whereas the role of inflammation in IBD dysplasia seems to be mainly critical to the initiation phase of the oncogenic process. This is consistent with previous studies that showed that an ‘immune tolerant’ but not ‘inflammatory or highly immunogenic phenotype’ is enriched in IBD compared to sporadic CRC^9,19^. We also show that in PSC the inflammatory transcriptional I2 signature may be a clinical predictor for the development of dysplasia in patients with PSC. Importantly, we observed the I2 signature in patients with PSC with no history of dysplasia, suggesting that this inflammation is not a response to dysplasia, but rather precedes it. Overall, the I2 signature can identify patients with PSC who need to be more closely monitored for dysplasia and who may require more aggressive therapies. A prospective study in which patients are classified as I2 or non-I2 and followed for right-sided dysplasia outcomes is warranted and would validate our results. We propose the use of the I2 PSC classifier model consisting of 81 genes (Fig. 6) as a surveillance tool to identify patients with PSC at higher risk of developing CRN.

Furthermore, our study implicates adaptive immunity in the development of CRN in patients with PSC. Indeed, the I2 signature is characterized by a clonally expanded IL-17A^+^FOXP3^+^ T cell and IgG-secreting plasma cell immune responses. The involvement of IL-17A^+^FOXP3^+^ DP T cells in colitis-associated cancers was previously suggested^21^. Our study suggests that DP T cells are significantly increased in PSC compared to IBD, and that they may have a distinct role in the progression of PSC dysplasia. The finding that DP cells have an activated and pathogenic helper T 17 (T_H_17) phenotype, suggests that they may be driving dysplasia because of the cytokines and factors that they produce. The expanded and mutated IgG-secreting plasma cells might also contribute to CRN by promoting the expansion of pathogenic T cells. Indeed, in HLA-associated diseases, B cell and T cell cross talk has been implicated in the amplification of pathogenic tissue destruction^32,33^. The relative expansion of IgG plasma cells compared to IgA may be the result of impaired class switching of IgG1 B cells to IgA, or may be due to a tissue environment that favors the differentiation of IgG B cells. Our data cannot distinguish between these two possibilities.

These findings, in combination with the strong HLA class II association, suggest that specific antigens may be driving inflammatory adaptive immune responses that promote CRN in PSC. If so, interventions that target this adaptive immune response, including targeting B cells, or removing the driving antigens could dramatically reduce the risk of CRN in PSC. Although the antigens are to be identified, some studies have pointed to bacteria as the source of antigens in PSC^34^ and CRN^35^. Small clinical studies on PSC cohorts have shown improvements in liver function tests and inflammation after antibiotic treatment^36–38^. We have generated immunoglobulin and TCRs that can be used to screen and identify potential bacterial antigens that can formally test the relationship of a specific taxon or taxa with dysplasia.

Finally, although the relationship between the intestinal and liver pathologies in PSC are unclear, it is possible that the mechanisms of intestinal inflammation also cause bile duct fibrosis, although such an investigation remains outstanding. Therefore, further investigation of the mechanisms of CRN in this study could not only lead to interventions that reduce rates of CRN in PSC, but also decrease rates of liver pathologies.

## Methods

### Patient enrollment and ethics

Enrollment of patients at UChicago Medicine (UCM), collection of samples and sample analysis were approved by the University of Chicago institutional review board (IRB) and performed under IRB protocol nos. 15573A and 13–1080. Samples collected at the Washington University School of Medicine were collected under the IRB no. 201111078. Samples collected at the Ichan School of Medicine at Mount Sinai were collected under GCO 14-0727.

Adults scheduled for a standard of care colonoscopy at UCM were screened for diagnosis and eligibility criteria for enrollment on a weekly basis. Exclusion criteria included: patients with active or chronic infections such as HIV, hepatitis B, hepatitis C or active, untreated *Clostridium difficile*; active infection with severe acute respiratory syndrome coronavirus 2; intravenous or illicit drug use such as cocaine, heroin or nonprescription methamphetamines; active use of blood thinners; severe comorbid diseases; patients on active cancer treatment; and patients who are pregnant. Approaching prospective patients was at the discretion of their treating physician and was not done in cases that would put patients at any increased risk, regardless of the reason. Patients were approached the day of their procedure and informed, written consent was obtained before the procedure. No financial compensation was provided to participants. The sex of each participant was self-reported; we took careful consideration to ensure that there was a balance of sexes across diagnosis groups. Sex was used as a covariate in the tissue transcriptional analysis that is the basis of all subsequent analyses. No sex-stratified analysis was performed because the proportion of patients identifying as female were included when comparing PSC to IBD (35% versus 38% without dysplasia and 50% versus 57% with dysplasia) This sex distribution is consistent with the known sex distribution within PSC (approximately 60% male). Race and ethnicity were both self-reported in our study and included as covariates in the tissue transcriptional analysis that is the basis of all subsequent analyses. There was no significant difference in the distribution of ethnic groups across patient groups (Extended Data Tables 1 and 2).

### Classification of patients into diagnosis groups

Patients were categorized as PSC, IBD or healthy (no diagnosis of PSC or IBD) individuals (HCs). Patients with IBD and PSC were further subclassified according to IBD type. Should a patient’s diagnosis change over the course of the study (for example, the subtype of IBD was rediagnosed as UC, when previously Crohn’s), the most recent diagnosis was used for all time points. Categorization of each patient into a diagnosis group was done after careful review of the patient’s medical health records and confirmation by an attending gastroenterologist. Patients were classified as PSC if records of a diagnosis of PSC could be found in the patient chart along with supporting liver imaging and liver function tests consistent with the diagnosis of PSC. A liver biopsy was not necessary to confirm a diagnosis of PSC as consistent with current practices.

Patients with IBD were enrolled only if they had a documented history of right-sided colitis before the procedure. Any patients without a diagnosis of PSC or IBD who were receiving screening colonoscopies for preventative cancer screening or diagnostic abnormalities such as diarrhea, were considered healthy individuals. All healthy individuals consented to the study who were determined to have signs of endoscopic or histological inflammation were excluded retrospectively from the study.

If the pathologist reported evidence of adenoma, low-grade dysplasia, high-grade dysplasia or adenocarcinoma, the patient was classified as having dysplasia. If the pathologist reported indefinite dysplasia or were unable to determine whether an abnormal lesion represented actual dysplasia or reactive changes due to inflammation, the patient was classified as indefinite for dysplasia. If no signs of bona fide or indefinite dysplasia were identified, the patient was classified as nondysplastic. Sporadic dysplasia was defined as the presence of dysplasia (typically an adenoma) in healthy individuals.

### Collection of patient clinical and demographic data

The demographic information collected included date of birth, sex and ethnicity. We also recorded the date of initial IBD and PSC diagnosis, the date of first incidence of dysplasia and the date of liver transplant. For each procedure, we recorded the date of the procedure; endoscopically and histologically scored inflammation in the right colon; location, stage and nature of dysplasia; endoscopically and histologically scored inflammation at the site of dysplasia; and all IBD-related or PSC-related medications currently taken by the patients, including immunosuppressants, biologics, antibiotics, steroids and ursodiol.

Endoscopically scored inflammation was based on the clinician’s evaluation of inflammation using the Mayo Endoscopic Subscore system^39^. The following scale was used: 0, no diagnostic abnormality or quiescent inflammation; 1, mild inflammation; 2, moderate inflammation; and 3, severe inflammation.

Histologically scored inflammation was based on the pathologist’s evaluation of the inflammation based on the histological criteria for grading of disease activity at UCM. The criteria are the following: 0, no diagnostic abnormality; 1, quiescent with features of chronicity (crypt distortion, shortening or drop-out, basal plasmacytosis, pyloric or Paneth cell metaplasia) in the absence of mild, moderate or severe activity; 2, mild with neutrophils present in the epithelium; 3, moderate with the neutrophils present in crypt lumen forming crypt abscesses; and 4, severe with erosion or ulceration of epithelium.

### Collection of tissue specimens

During the colonoscopy, the endoscopist collected 8–10 tissue biopsies using 2.8-mm or 3.2-mm forceps at 10 cm distal to the ileocecal valve. One of these biopsies was placed immediately into RNAprotect (QIAGEN) and the remaining biopsies were placed into Roswell Park Memorial Institute (RPMI) 1640 (Thermo Fisher Scientific). Samples were immediately transported on ice to the laboratory for processing, according to the protocols outlined below.

### Tissue biopsy RNA-seq

The tissue biopsy in RNAprotect was stored at 4 °C for 48–72 h, RNAprotect was removed and the biopsy stored at −80 °C until tissue processing. Biopsies stored at −80 °C were thawed on ice and transferred to Sarstedt tubes (Thermo Fisher Scientific) containing 350 μl RLT Plus buffer (QIAGEN) supplemented with 1% 2-mercaptoethanol (Thermo Fisher Scientific) and equal quantities of 1.0-mm and 0.5-mm zirconium oxide beads (one scoop each, Next Advance). Biopsies were bead beat three times for 1 min at a setting of 9 on a Bullet Blender 24 (Next Advance), with 1 min of cooling on ice between each beating. Lysates were processed using the AllPrep DNA/RNA/miRNA Universal Kit (QIAGEN); 500 ng of purified RNA was used as input in the TruSeq Stranded mRNA Library Prep kit (Illumina) to generate sample libraries according to the manufacturer’s specifications. Libraries were multiplexed and sequenced at a depth of 20 million reads per sample (50 bp, single-read) on a HiSeq 4000 sequencer.

### Lamina propria lymphocyte isolation

Colonic lymphocytes were isolated via mechanical disruption and enzymatic digestion. Briefly, colonic biopsies were shaken twice at 250 rpm for 30 min at 37 °C in 7 ml RPMI 1640 supplemented with 1% dialyzed FCS (Biowest), 2 mM EDTA (Corning) and 1.5 mM MgCl_2_ (Thermo Fisher Scientific). This fraction was discarded. Subsequently, tissue was digested in two sequential shakes at 250 rpm at 37 °C for 30 min in 15 ml RPMI 1640 supplemented with 20% fetal bovine serum and 1 mg ml^−1^ collagenase type IV, from *Clostridium histolyticum* (Sigma-Aldrich). After each digestion, the solution was filtered, centrifuged and then combined for downstream experimentation. This fraction was considered the lamina propria fraction.

### Surface flow cytometry and FACS

Cells were stained for 15 min on ice using LIVE/DEAD Fixable Aqua (1:50, Thermo Fisher Scientific) diluted in PBS (Thermo Fisher Scientific), washed with PBS supplemented 2% FCS and subsequently stained in an antibody cocktail for 25 min at 4 °C. The following directly conjugated antibodies were used to identify cell surface markers: mouse anti-human CD45-BV711 at 1:500 dilution (clone HI30, catalog no. 564357, BD Biosciences); mouse anti-human CD3-PE-Cy7 at 1:100 dilution (clone UCHT1, catalog no. 300420, BioLegend); mouse anti-human TCR α/β-BV421 at 1:20 dilution (clone IP26, catalog no. 306722, BioLegend); mouse anti-human CD4-BV510 at 1:50 dilution (clone SK3, catalog no. 562970, BD Biosciences), mouse anti-human CD8-BUV496 at 1:50 dilution (RPA-T8, BD Biosciences 612942); mouse anti-human CD19-PE at 1:50 dilution (clone HIB19, catalog no. 561741, BD Biosciences); mouse anti-human CD27-BV605 at 1:50 dilution (clone O323, catalog no. 302830, BioLegend); and mouse anti-human CD38-PerCP-Cy5.5 at 1:100 dilution (clone HIT2, catalog no. 303522, BioLegend). Cells were washed with PBS and 2% FCS, resuspended into PBS and 2% FCS, and subsequently run on a BD FACSAria Fusion Flow Cytometer to sort and purify the populations of interest. CD4 T cells (CD45^+^LIVE/DEAD^negative^ > forward scatter (FSC) versus side scatter (SSC) > singlets > CD3^+^CD19^negative^ > CD4^+^ CD8^negative^) and plasma cells (CD45^+^ LIVE/DEAD^negative^ > FSC versus SSC > singlets > CD3^negative^ > CD38^+^CD27^+^) from the lamina propria fraction were sorted into 600 μl of RPMI 1640 supplemented with 10% FCS and 1% penicillin/streptomycin (Thermo Fisher Scientific) for downstream experimentation including 10x Genomics sequencing and ELISpot. All flow cytometry data were analyzed using FlowJo v.10.7.2 (FlowJo LLC).

### ELISpot assay

Preceding the isolation of plasma cells, flat-bottom 96-well polystyrene plates (Thermo Fisher Scientific) were coated with polyclonal goat anti-human IgA, IgG and IgM antibodies (KPL, catalog no. 5210-0160, SeraCare) at a concentration of 5 μg ml^−1^, diluted in PBS (100 μl per well) and incubated at 4 °C for a minimum of 24 h. Coated plates were washed three times with PBS and 0.05% Tween 20 (Bio-Rad Laboratories) and then three times with PBS. Coated wells were then blocked with RPMI 1640 supplemented with 10% FCS and 1% penicillin/streptomycin at 37 °C for a minimum of 2 h. After FACS sorting, an equal number of plasma cells were diluted serially at 1:2 and left to incubate at 37 °C overnight. Cells were removed from the plate and the wells were washed three times with PBS and 0.05% Tween 20 and then three times with PBS. Wells were incubated with Biotin-conjugated polyclonal goat anti-human IgA, IgG or IgM (catalog nos. 2050-08, 2040-08 and 2020-08, respectively, Southern Biotech) at a concentration of 1 μg ml^−1^ at room temperature in the dark for 2 h. Subsequently, wells were washed three times with PBS and 0.05% Tween 20, three times with PBS and incubated in streptavidin-alkaline phosphotase (Southern Biotech) at a dilution of 1:500 for 2 h at room temperature in the dark. The wells were then washed three times in each PBS and 0.05% Tween 20 and PBS; the substrate NBT/BCIP (Thermo Fisher Scientific) was applied until individual spots were visible (fewer than 5 min) and the reaction was halted using room temperature tap water. Plates were left to dry upside down in the dark, after which images were captured using a CTL Analyzer (ImmunoSpot) and spots were quantified manually in ImageJ (NIH).

### Phorbol myristate acetate and ionomycin stimulation assay

Lamina propria cells were suspended in RPMI 1640 medium supplemented with 10% FCS, 1% penicillin/streptomycin, 1 pg ml^−1^ phorbol myristate acetate (Sigma-Aldrich), 1.5 ng ml^−1^ ionomycin calcium salt (Sigma-Aldrich), 0.15% GolgiPlug (BD Bioscience) and 0.3% GolgiStop (BD Bioscience) in a volume of 500 μl in a polystyrene flat-bottom, 24-well plate (Thermo Fisher Scientific). Cells were incubated at 37 °C for 3 h after which they were washed twice with ice-cold RPMI 1640 medium supplemented with 10% FCS and 1% penicillin/streptomycin. Cells were stained for viability and subsequently surface markers as for FACS, after which cells were fixed and permeabilized in a 1:4 solution of Fixation/Permeabilization Concentrate and Fixation/Diluent (eBioscience) for 1 h at 4 °C in the dark. Cells were washed twice with a 1:10 dilution of Permeabilization Buffer Solution (eBioscience) in nuclease-free water (Thermo Fisher Scientific) and subsequently stained for intracellular markers for 1 h at room temperature in the dark. The following directly conjugated antibodies were used to identify intracellular markers: mouse anti-human CD45-BV711 at 1:500 dilution; mouse anti-human TCR α/β-BV421 at 1:20 dilution; mouse anti-human CD4-BV510 at 1:50 dilution; mouse anti-human CD8-BUV496 at 1:50 dilution; mouse anti-human IFNγ-PE at 1:100 dilution (clone 4S.B3, catalog no. 12-7319-82, Thermo Fisher Scientific); mouse anti-human TNFα-FITC at 1:100 dilution (clone Mab11, catalog no. 502906, BioLegend); mouse anti-human IL-17A-APC at 1:50 dilution (clone BL168, catalog no. 512334, BioLegend); and rat anti-human FOXP3-PE-Cy7 at 1:20 dilution (clone PCH101, catalog no. 25-4776-42, Thermo Fisher Scientific). Cells were subsequently washed and passed on either a BD LSRFortessa flow cytometer or a Cytek Aurora flow cytometer. All flow cytometry data were analyzed using FlowJo v.10.7.2 (FlowJo LLC).

### scRNA-seq

Cells were centrifuged and resuspended to a final concentration in RPMI 1640 medium supplemented with 10% FCS and 1% penicillin/streptomycin, and the suspensions were loaded into a Chromium Controller (10x Genomics) under conditions to generate an anticipated yield of 1,000–10,000, depending on the yield of cells from tissue. Single-cell 5′ RNA-seq libraries and V(D)J libraries were generated for each sample according to the manufacturer’s instructions (Chromium Single Cell 5′ Library Construction Kit V1 Chemistry, Single Cell V(D)J Enrichment Kit for Human T cells, and Single Cell V(D)J Enrichment Kit for Human B cells, all from 10x Genomics). 5′ libraries were sequenced to a minimum depth of 50,000 reads per cell for 5′ gene expression libraries, or 5,000 reads per cell for V(D)J libraries, on an Illumina NovaSeq 6000.

### Bulk RNA-seq analysis

All bulk RNA-seq samples were processed using a standard workflow based on the GenPipes framework^40^. Specifically, the stringtie type rnaseq pipeline was used. Reads were first trimmed using Trimmomatic^41,42^ v.0.40. Trimmed reads were aligned to the GRCh38 human reference genome using STAR aligner^42^ v.2.7.10b according to a two-pass mapping protocol. Alignments were then sorted and filtered for duplicates using the markduplicates function of Picard v.3.0.0 (http://broadinstitute.github.io/picard/)^43^. Gene-level read counts for downstream processing were calculated from spliced alignments using HTseq count^44^ v.0.11.1.

### Dimensionality reduction and clustering in nondysplastic samples

The normalized (log_2_ count per million reads (CPM)) expression matrix for the nondysplastic samples was corrected for batch effect and we selected the top 3,000 most variable genes by modeling the mean variance relationship using the FindVariableFeatures function from the Seurat package^45–48^ v.4.0.0.0. Next, we calculated the principal components by sample, for which we selected the first 40 principal components because they explain at least 70% of the complete variance. These 40 principal components were then used as a distance matrix to perform hierarchical clustering from which we selected four biologically relevant clusters: U1, U2, I1 and I2. All statistical analyses involving dimensionality reduction and clustering were performed using R v.4.0.3.

### Differential expression and GSEA

Counts derived from the alignment were filtered for lowly expressed transcripts (median > 5). Furthermore, we included only protein-coding genes and TCR and Ig receptors, resulting in a total of 15,146 genes. On this set of genes, we detected DEGs either across diagnosis or cluster by fitting a linear model to the log_2_ CPM using the limma package^49^ v.3.46.0. In every contrast, we included as covariates sex, age and batch of sequencing.

We performed GSEA using the gseaGO function from clusterProfiler^50,51^ v.3.0.4 over the log_2_ fold changes in expression between contrasts (cluster I2 versus I1).

To detect GOs enriched in defined sets of genes, such as I2 PSC genes (I2 PSC versus I2 IBD contrast, adjusted *P* < 0.05, log_2_ fold change > 0). We performed over-enrichment analysis using the enrichGO function from clusterProfiler v.3.0.4.

### Prediction of cluster assignment in dysplastic samples

To assign a cluster (U1, U2, I1 and I2) to dysplastic samples, we constructed a classifier using an eNet model^52^, which is a regularized regression approach. We decreased the potential noise within cluster assignment errors by calculating the cluster silhouette for each sample; we selected only samples with a positive silhouette score. We used a core cluster of samples to detect DEGs between the U2, I1 and I2 clusters and used all DEGs (adjusted *P* < 0.05) in at least one contrast as the initial set of features to construct the eNet model. Next, we partitioned the cohort of core samples into a training set of 70% of all samples and a test set with the rest. To select the penalization score for eNet, we used a 10× cross validation within the training cohort. The resulting classification model to predict I2 cluster adscription consisted of 81 genes with nonzero coefficients. The I2 model predicted with 100% accuracy the out-of-sample test cohort (AUC = 1).

### Transcriptional analysis of CD4 T cells

FASTQ files were processed into gene count matrices using Cell Ranger v.3.1.0 (https://support.10xgenomics.com/single-cell-gene-expression/software/pipelines/latest/what-is-cell-ranger) and the GRCh38 (https://www.ncbi.nlm.nih.gov/assembly/GCF_000001405.26/) transcriptome. Analysis was centered on the Seurat framework. Filtration included the removal of plasma cells from some samples and cells with a mitochondrial read percentage greater than 50%. We dropped samples PSC28D and PSC40D entirely due to low T cell counts. Datasets were integrated using the SCTransform protocol. Specifically, SCTransform was run on each sample while regressing the mitochondrial read percentage as a covariate. Integration was performed using 20,000 genes followed by dimensionality reduction runPCA (using 20 principle components for all dependent analysis) and runUMAP. After dimensionality reduction, unsupervised clustering was performed using FindNeighbors and FindClusters (resolution of 1). To define T cell subpopulations, we used a calibration strategy with corresponding flow cytometry data as a reference (Extended Data Fig. 4).

To perform differential gene expression analysis, we used a pseudobulking strategy. First, genes were filtered to have log CPM > 0.01. Next, scran factor normalization was performed using the computeSumFactors function from the scran R package^53^. Cells with size factors between 0.125 and 8 were preserved. Pseudobulk means were then calculated from the log counts as the per-gene mean within each pseudobulk grouping. Pseudobulk means were used as input into an limma voom differential testing pipeline similar to those employed in bulk. Variance stabilization was performed using the limma voom function voomWithQualityWeights and model fitting was performed using the limma voom functions lmfit and eBayes. Resulting differential expression statistics were extracted using the topTable function.

### Repertoire analysis of CD4 T cells

The binary base call output from sequencing was put through the Cell Ranger mkfastq pipeline to generate FASTQ files, which were subsequently put through Cell Ranger vdj to generate full-length TCR sequences (https://support.10xgenomics.com/single-cell-vdj/software/pipelines/latest/using/vdj). Full-length TCR sequences were processed using IMGT/V-QUEST^54,55^ to identify productive sequences, determine V, D and J gene use, and identify the CDR3. Nonproductive sequences, and sequences with the same cellular barcode, were filtered from the analysis. TCRs were matched to gene expression profiles by barcodes and all subsequent analyses were performed according to cell type. CDR3 amino acid sequences were trimmed from both ends to the leftmost and rightmost amino acid with a mutation within its codon (silent or missense). Trimmed amino acids from *IL17A*^+^*FOXP3*^+^ CD4 T cells were queried for potential motifs using the Sensitive, Thorough, Rapid, Enriched Motif Elicitation web-based software (https://meme-suite.org/meme/doc/streme.html)^56^, using *IL17A* and *FOXP3*^*+*^ SP CDR3 cells as a control. The proportion of cells containing the motif were then calculated manually.

### Repertoire analysis of plasma cells

The binary base call output from sequencing was run through the Cell Ranger mkfastq pipeline to generate FASTQ files, which were subsequently run through Cell Ranger vdj to generate full-length Ig sequences. IMGT/V-QUEST v.3.6.0 (https://www.imgt.org/IMGTindex/V-QUEST.php) was used to identify productive sequences, determine V, D, and J gene use, and identify the CDR3. Nonproductive sequences and sequences with the same cellular barcode were filtered from the analysis. Partis v.0.15.0 with default settings was used to simultaneously identify sets of sequences descended from the same naive B cell and determine the sequence and germline immunoglobulin genes used by each clone’s naive ancestor. IgPhyML^57,58^ v.1.1.0 was used to build clones’ phylogenetic trees by jointly optimizing tree topology and the parameters of a codon substitution model that incorporates variation in the mutability of nucleotide motifs in immunoglobulin genes. We manually verified that all the heavy chains within the top clones used the same light chain. Those that did not were removed from the clone and clonal size was readjusted. Custom code (https://github.com/cobeylab/psc_repertoire) was used for subsequent computational analyses.

For the entire sequence and separately for CDR3, the average amino acid divergence was computed between each sequence and the inferred naive ancestor (to estimate average divergence from the clone’s ancestor) and for all pairs of sequences in a clone (to estimate standing diversity within clones at the time they were sampled). These analyses were conducted for the top clone in each dataset, including multiple clones in case of ties.

### Patient genotyping and HLA imputation

The DNA of patients was genotyped using the Illumina Infinium global screening array v.1.0, with accompanying manifest file A5. Per patient, 200 ng of DNA was used for hybridization; visualization was performed using the Illumina iScan. Results were exported using GenomeStudio. Genotype calling was performed using opticall v.0.8.1; all samples reported a call rate greater than 98%, and genotypes with a call rate below 95% were removed. Furthermore, rare variants (minor allele frequency < 0.01) and variants that do not follow the Hardy–Weinberg equilibrium (*P* < 0.0001) were removed from further analysis. Genotypes where then prepared for imputation according to the guidelines and toolbox from the Michigan imputation server (https://imputationserver.sph.umich.edu/index.html#!) to be matched to the genome assembly GRCh37/hg19. Genotypes from chromosome 6 where then used to impute the HLA region using the four-digit multiethnic HLA reference panel v.1. We then used the imputed four-digit HLA annotations to infer the HLA haplotypes for each patient.

### Time to dysplasia Kaplan–Meier analysis

The medical records of each patient with IBD and PSC was probed to determine the date of diagnosis of colitis, last date of follow-up at UCM and the date of first incidence of right-sided or non-right-sided dysplasia (if applicable). Right-sided dysplasia was dysplasia occurring in the cecum, ascending colon or hepatic flexure. Non-right-sided dysplasia was considered dysplasia occurring in the transverse colon, splenic flexure, descending colon, sigmoid colon or rectum. Right-sided dysplasia and non-right-sided dysplasia were considered independent events. We calculated the time from colitis diagnosis to right-sided dysplasia for each individual patient with a history of right-sided dysplasia or to the most recent colonoscopy at UCM for patients with no documented right-sided dysplasia. We stratified samples into two groups: I2 and non-I2. We defined I2 as any samples for which an I2 inflammatory profile was ever detected in any of their visits, including visits after or during the first diagnosis of right-sided dysplasia. We then evaluated the difference in time to develop right-sided dysplasia from their first colitis-related diagnosis using the Kaplan–Meier estimator, using the survminer package v.0.4.8 (https://CRAN.R-project.org/package=survminer), for both patients with PSC and patients with IBD. The same process was then repeated with non-right-sided dysplasia as the outcome.

### Statistics and reproducibility

No statistical method was used to predetermine sample size due to the rare nature of PSC. Samples from patients with an unclear diagnosis were excluded retrospectively from the study. If the same patient was sampled at multiple visits, only the first sample was used in the analysis of the tissue RNA-seq. For the subsequent analyses, if the same patient was sampled on multiple visits, only a single sample was included per transcriptional cluster. Samples that did not pass quality control for transcriptional analysis were excluded as described in the methods above. The experiments were not randomized. The investigators were not blinded to allocation during the experiments and outcome assessment.

### Reporting summary

Further information on research design is available in the Nature Portfolio Reporting Summary linked to this article.

## Online content

Any methods, additional references, Nature Portfolio reporting summaries, source data, extended data, supplementary information, acknowledgements, peer review information; details of author contributions and competing interests; and statements of data and code availability are available at 10.1038/s41591-023-02372-x.

### Supplementary information

Reporting Summary

## Figures and Tables

**Extended Data Table 1:**
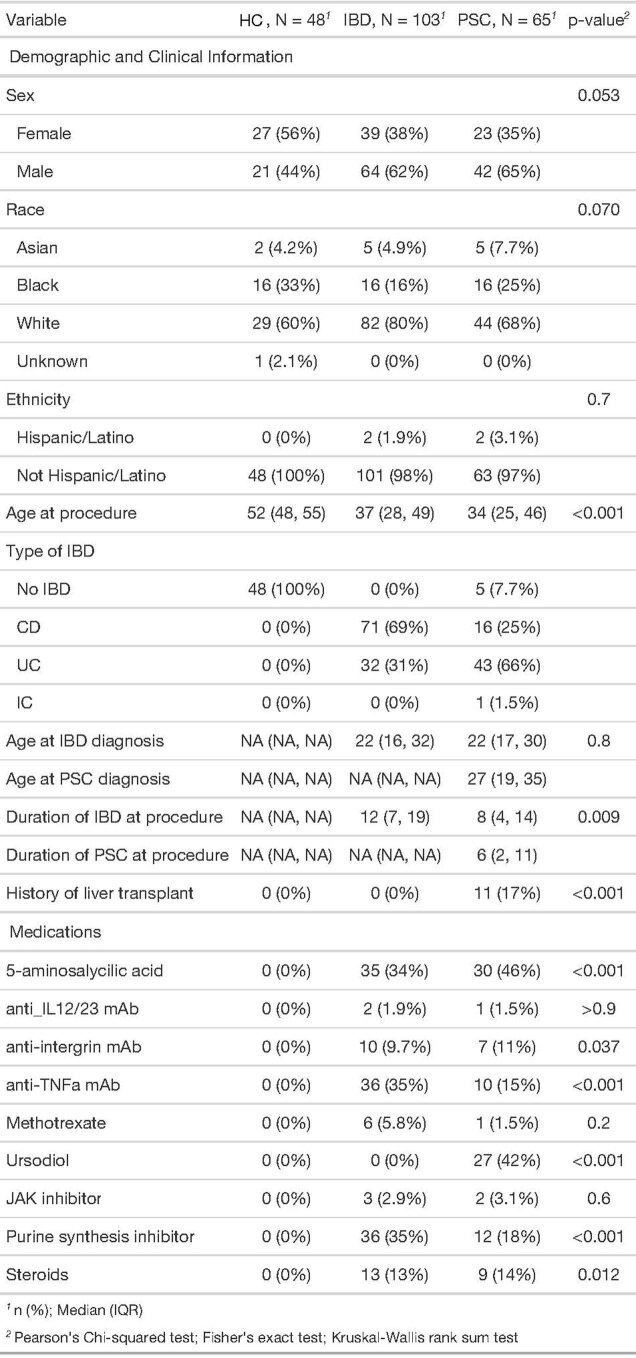
Clinical and demographic information for patients with no history of dysplasia analyzed in Fig. 1

Clinical and demographic information for patients with no history of dysplasia analyzed in Fig. 1. Sex and race were self-reported. All ages and durations are calculated in years.

**Extended Data Table 2:**
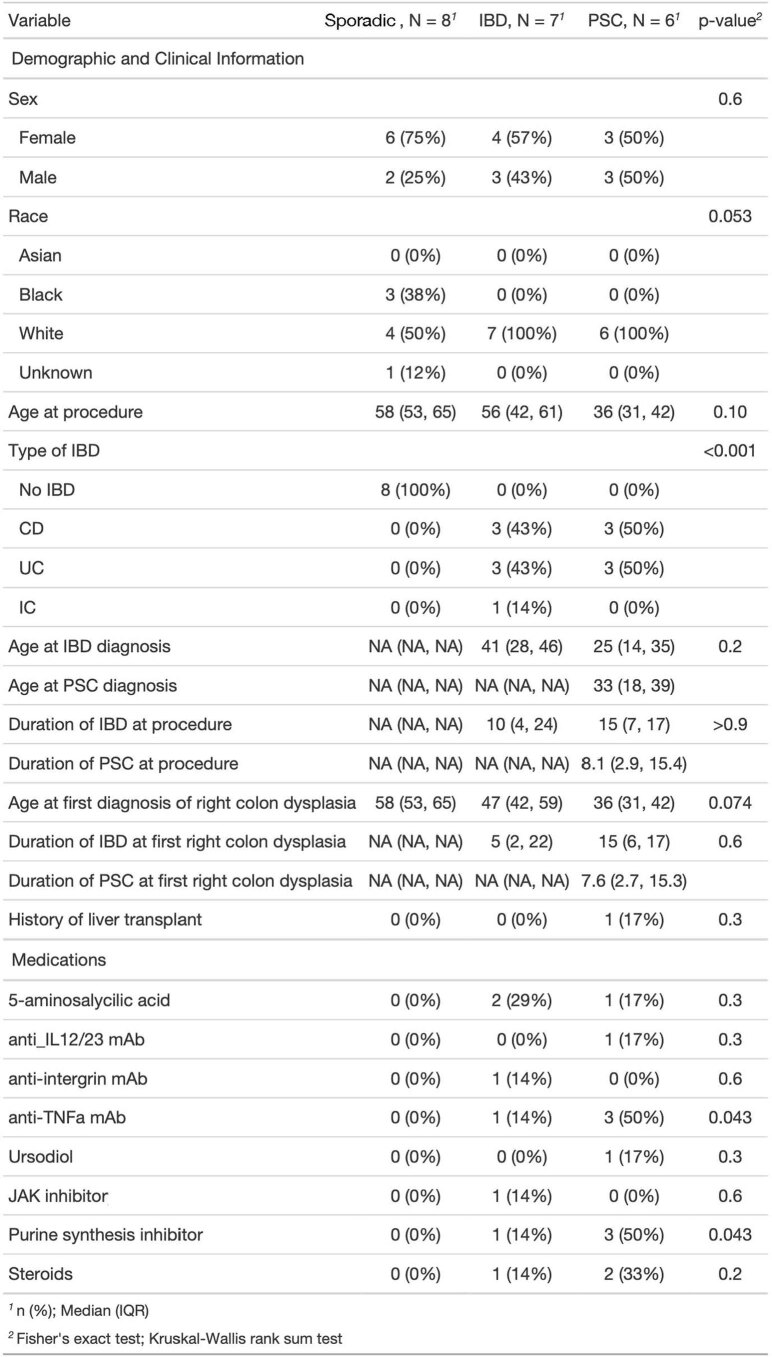
Clinical and demographic information for patients with active right-sided dysplasia analyzed in Fig. 2

Clinical and demographic information for patients with active dysplasia analyzed in Fig. 2. Sex and race were self-reported. All ages and durations are calculated in years.

**Extended Data Table 3:**
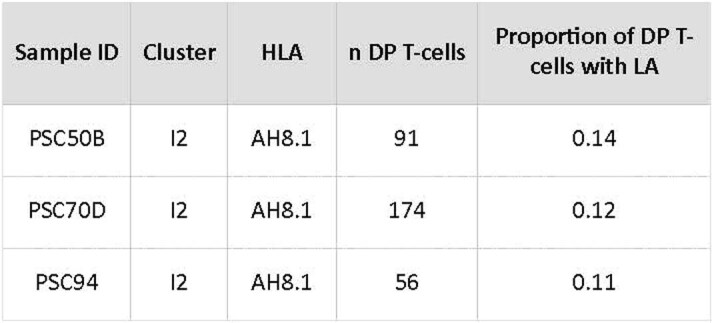
Patients imputed HLA haplotypes and percentage of *IL17A*^+^*FOXP3*^+^ DP CD4 T cells with LA

The sample ID is the unique patient ID. Cluster is the transcriptional cluster to which the sample belongs. HLA denotes the HLA haplotype of the patient. N DP T cells denotes the number of DP T cells analyzed by single-cell RNA sequencing in this patient. The last column denotes the proportion of DP cells containing the ‘LA’ motif.

## Data Availability

Raw expression data from both bulk (gut biopsies) and single cells from purified CD4^+^ T cells and plasma cells are deposited in the Gene Expression Omnibus (accession no. GSE230524 for gut biopsy RNA-seq and accession no. GSE230569 for CD4 T cell and plasma cell single-cell gene expression sequencing and repertoire sequencing). Process flow cytometry, ELISpot and clinical meta-data can be accessed at the Zenodo repository (10.5281/zenodo.7857026). Individual-level data are available from these repositories without time limitation: GRCh38 can be accessed at https://www.ncbi.nlm.nih.gov/assembly/GCF_000001405.26/; GRCh37/hg19 can be accessed at https://www.ncbi.nlm.nih.gov/assembly/GCF_000001405.13/.

## Code availability

Alongside processed data, the code relevant to the analysis and figures in the manuscript was deposited in the Zenodo repository (10.5281/zenodo.7857026). Code related to the Ig analysis can be accessed at https://github.com/cobeylab/psc_repertoire.

## References

[1] Bowlus CL, Li C-S, Karlsen TH, Lie BA, Selmi C (2010). Primary sclerosing cholangitis in genetically diverse populations listed for liver transplantation: unique clinical and human leukocyte antigen associations. Liver Transpl..

[2] Lee Y-M, Kaplan MM (1995). Primary sclerosing cholangitis. N. Engl. J. Med..

[3] Fausa O, Schrumpf E, Elgjo K (1991). Relationship of inflammatory bowel disease and primary sclerosing cholangitis. Semin. Liver Dis..

[4] Shah SC (2018). High risk of advanced colorectal neoplasia in patients with primary sclerosing cholangitis associated with inflammatory bowel disease. Clin. Gastroenterol. Hepatol..

[5] Broomé U, Löfberg R, Veress B, Eriksson LS (1995). Primary sclerosing cholangitis and ulcerative colitis: evidence for increased neoplastic potential. Hepatology.

[6] Rutter M (2004). Severity of inflammation is a risk factor for colorectal neoplasia in ulcerative colitis. Gastroenterology.

[7] Lutgens MWMD (2013). Declining risk of colorectal cancer in inflammatory bowel disease: an updated meta-analysis of population-based cohort studies. Inflamm. Bowel Dis..

[8] Grivennikov SI, Greten FR, Karin M (2010). Immunity, inflammation, and cancer. Cell.

[9] Shah SC, Itzkowitz SH (2022). Colorectal cancer in inflammatory bowel disease: mechanisms and management. Gastroenterology.

[10] Beaugerie L, Itzkowitz SH (2015). Cancers complicating inflammatory bowel disease. N. Engl. J. Med..

[11] Ji S-G (2017). Genome-wide association study of primary sclerosing cholangitis identifies new risk loci and quantifies the genetic relationship with inflammatory bowel disease. Nat. Genet..

[12] Loftus EV (2005). PSC-IBD: a unique form of inflammatory bowel disease associated with primary sclerosing cholangitis. Gut.

[13] Joo M (2009). Pathologic features of ulcerative colitis in patients with primary sclerosing cholangitis: a case-control study. Am. J. Surg. Pathol..

[14] Shetty K, Rybicki L, Brzezinski A, Carey WD, Lashner BA (1999). The risk for cancer or dysplasia in ulcerative colitis patients with primary sclerosing cholangitis. Am. J. Gastroenterol..

[15] Claessen MMH (2009). More right-sided IBD-associated colorectal cancer in patients with primary sclerosing cholangitis. Inflamm. Bowel Dis..

[16] James KR (2020). Distinct microbial and immune niches of the human colon. Nat. Immunol..

[17] Moum B, Ekbom A, Vatn MH, Elgjo K (1999). Change in the extent of colonoscopic and histological involvement in ulcerative colitis over time. Am. J. Gastroenterol..

[18] Jiang X, Karlsen TH (2017). Genetics of primary sclerosing cholangitis and pathophysiological implications. Nat. Rev. Gastroenterol. Hepatol..

[19] Guinney J (2015). The consensus molecular subtypes of colorectal cancer. Nat. Med..

[20] Itzkowitz SH (2006). Molecular biology of dysplasia and cancer in inflammatory bowel disease. Gastroenterol. Clin. North Am..

[21] Keerthivasan S (2014). β-Catenin promotes colitis and colon cancer through imprinting of proinflammatory properties in T cells. Sci. Transl. Med..

[22] Grishkan IV, Ntranos A, Calabresi PA, Gocke AR (2013). Helper T cells down-regulate CD4 expression upon chronic stimulation giving rise to double-negative T cells. Cell. Immunol..

[23] Wang H (2015). Granzyme M expressed by tumor cells promotes chemoresistance and EMT in vitro and metastasis in vivo associated with STAT3 activation. Oncotarget.

[24] Sloot YJE, Smit JW, Joosten LAB, Netea-Maier RT (2018). Insights into the role of IL-32 in cancer. Semin. Immunol..

[25] Lee Y (2012). Induction and molecular signature of pathogenic T_H_17 cells. Nat. Immunol..

[26] Henriksen EKK (2017). HLA haplotypes in primary sclerosing cholangitis patients of admixed and non-European ancestry. HLA.

[27] Farstad IN, Carlsen H, Morton HC, Brandtzaeg P (2000). Immunoglobulin A cell distribution in the human small intestine: phenotypic and functional characteristics. Immunology.

[28] Landsverk OJB (2017). Antibody-secreting plasma cells persist for decades in human intestine. J. Exp. Med..

[29] Horns F, Vollmers C, Dekker CL, Quake SR (2019). Signatures of selection in the human antibody repertoire: selective sweeps, competing subclones, and neutral drift. Proc. Natl Acad. Sci. USA.

[30] Donaldson GP, Lee SM, Mazmanian SK (2015). Gut biogeography of the bacterial microbiota. Nat. Rev. Microbiol..

[31] Calderó J (1989). Regional distribution of glycoconjugates in normal, transitional and neoplastic human colonic mucosa. A histochemical study using lectins. Virchows Arch. A Pathol. Anat. Histopathol..

[32] Jabri B, Sollid LM (2009). Tissue-mediated control of immunopathology in coeliac disease. Nat. Rev. Immunol..

[33] Lejeune T, Meyer C, Abadie V (2021). B lymphocytes contribute to celiac disease pathogenesis. Gastroenterology.

[34] Nakamoto N (2019). Gut pathobionts underlie intestinal barrier dysfunction and liver T helper 17 cell immune response in primary sclerosing cholangitis. Nat. Microbiol..

[35] Dejea CM (2018). Patients with familial adenomatous polyposis harbor colonic biofilms containing tumorigenic bacteria. Science.

[36] Davies YK (2008). Long-term treatment of primary sclerosing cholangitis in children with oral vancomycin: an immunomodulating antibiotic. J. Pediatr. Gastroenterol. Nutr..

[37] Tabibian JH (2013). Randomised clinical trial: vancomycin or metronidazole in patients with primary sclerosing cholangitis—a pilot study. Aliment. Pharmacol. Ther..

[38] De Chambrun GP (2018). Oral vancomycin induces sustained deep remission in adult patients with ulcerative colitis and primary sclerosing cholangitis. Eur. J. Gastroenterol. Hepatol..

[39] Lobatón T (2015). The Modified Mayo Endoscopic Score (MMES): a new index for the assessment of extension and severity of endoscopic activity in ulcerative colitis patients. J. Crohns Colitis.

[40] Bourgey M (2019). GenPipes: an open-source framework for distributed and scalable genomic analyses.. Gigascience.

[41] Bolger AM, Lohse M, Usadel B (2014). Trimmomatic: a flexible trimmer for Illumina sequence data. Bioinformatics.

[42] Dobin A (2013). STAR: ultrafast universal RNA-seq aligner.. Bioinformatics.

[43] Broad Institute. Picard Toolkit 2019 version 3.0.0. https://broadinstitute.github.io/picard/ (2019).

[44] Anders S, Pyl PT, Huber W (2015). HTSeq—A Python framework to work with high-throughput sequencing data.. Bioinformatics.

[45] Hao Y (2021). Integrated analysis of multimodal single-cell data.. Cell.

[46] Stuart T (2019). Comprehensive integration of single-cell data. Cell.

[47] Butler A, Hoffman P, Smibert P, Papalexi E, Satija R (2018). Integrating single-cell transcriptomic data across different conditions, technologies, and species. Nat. Biotechnol..

[48] Satija R, Farrell JA, Gennert D, Schier AF, Regev A (2015). Spatial reconstruction of single-cell gene expression data. Nat. Biotechnol..

[49] Ritchie ME (2015). limma powers differential expression analyses for RNA-sequencing and microarray studies. Nucleic Acids Res..

[50] Wu T (2021). clusterProfiler 4.0: a universal enrichment tool for interpreting omics data. Innovation.

[51] Yu G, Wang L-G, Han Y, He Q-Y (2012). clusterProfiler: an R package for comparing biological themes among gene clusters. OMICS.

[52] Zou H, Hastie T (2005). Regularization and variable selection via the elastic net. J. R. Stat. Soc. Ser. B Stat. Methodol..

[53] Lun ATL, McCarthy DJ, Marioni JC (2016). A step-by-step workflow for low-level analysis of single-cell RNA-seq data with Bioconductor. F1000Res..

[54] Brochet X, Lefranc M-P, Giudicelli V (2008). IMGT/V-QUEST: the highly customized and integrated system for IG and TR standardized V-J and V-D-J sequence analysis. Nucleic Acids Res..

[55] Giudicelli V, Brochet X, Lefranc M-P (2011). IMGT/V-QUEST: IMGT standardized analysis of the immunoglobulin (IG) and T cell receptor (TR) nucleotide sequences. Cold Spring Harb. Protoc..

[56] Bailey TL (2021). STREME: accurate and versatile sequence motif discovery. Bioinformatics.

[57] Hoehn KB (2019). Repertoire-wide phylogenetic models of B cell molecular evolution reveal evolutionary signatures of aging and vaccination. Proc. Natl Acad. Sci. USA.

[58] Hoehn KB, Lunter G, Pybus OG (2017). A phylogenetic codon substitution model for antibody lineages. Genetics.

