# Supplementary material for: Antigen-driven colonic inflammation is associated with development of dysplasia in primary sclerosing cholangitis
Source: Nat Med. 2023 Jun 15;29(6):1520–9. doi: 10.1038/s41591-023-02372-x (PMC10287559; doi:10.1038/s41591-023-02372-x)
Supplement: Supplementary file 1 — Reporting Summary [file 41591_2023_2372_MOESM1_ESM.pdf]

nature portfolio

Corresponding author(s): Bana Jabri, MD PhD
Luis Barreiro, PhD

Last updated by author(s): Apr 23, 2023

Reporting Summary

Nature Portfolio wishes to improve the reproducibility of the work that we publish. This form provides structure for consistency and transparency in reporting. For further information on Nature Portfolio policies, see our Editorial Policies and the Editorial Policy Checklist.

Statistics

For all statistical analyses, confirm that the following items are present in the figure legend, table legend, main text, or Methods section.

n/a	Confirmed	
☐	☒	The exact sample size (*n*) for each experimental group/condition, given as a discrete number and unit of measurement
☐	☒	A statement on whether measurements were taken from distinct samples or whether the same sample was measured repeatedly
☐	☒	The statistical test(s) used AND whether they are one- or two-sided<br>*Only common tests should be described solely by name; describe more complex techniques in the Methods section.*
☐	☒	A description of all covariates tested
☐	☒	A description of any assumptions or corrections, such as tests of normality and adjustment for multiple comparisons
☐	☒	A full description of the statistical parameters including central tendency (e.g. means) or other basic estimates (e.g. regression coefficient) AND variation (e.g. standard deviation) or associated estimates of uncertainty (e.g. confidence intervals)
☐	☒	For null hypothesis testing, the test statistic (e.g. *F*, *t*, *r*) with confidence intervals, effect sizes, degrees of freedom and *P* value noted<br>*Give P values as exact values whenever suitable.*
☒	☐	For Bayesian analysis, information on the choice of priors and Markov chain Monte Carlo settings
☒	☐	For hierarchical and complex designs, identification of the appropriate level for tests and full reporting of outcomes
☐	☒	Estimates of effect sizes (e.g. Cohen's *d*, Pearson's *r*), indicating how they were calculated

Our web collection on statistics for biologists contains articles on many of the points above.

Software and code

Policy information about availability of computer code

Data collection	All flow cytometry data was collected using either BD LSRFortessa, Cytek Aurora, Or BD FACSAria Fusion flow cytometers. All single cell capture was performed on a 10x Genomics Chromium Controller. All sequencing was performed on an Illumina HiSeq4000 or Illumina NovaSeq6000.
Data analysis	Software used to analyze the data include: Trimmomatic (v 0.40), STAR (v 2.7.10b), Picard (v 3.0.0), HTseq count (v 0.11.1), Seurat (v 4.0.0.0), R (v 3.6.3 or above), Limma (v 3.46.0), clusterProfiler (v 3.0.4), Cellranger (v 3.1.0), STREME (v 5.5.2), IMGT/V-QUEST (v 3.6.0), IgPhyML (v1.1.0), Genomestudio (v 0.8.1), survminer (v0.4.8). Custom code related to the analysis is available at https://github.com/cobeylab/psc_repertoire and on Zenodo (DOI: 10.5281/zenodo.7857026).

For manuscripts utilizing custom algorithms or software that are central to the research but not yet described in published literature, software must be made available to editors and reviewers. We strongly encourage code deposition in a community repository (e.g. GitHub). See the Nature Portfolio guidelines for submitting code & software for further information.

Data

Policy information about availability of data

All manuscripts must include a data availability statement. This statement should provide the following information, where applicable:

- Accession codes, unique identifiers, or web links for publicly available datasets
- A description of any restrictions on data availability
- For clinical datasets or third party data, please ensure that the statement adheres to our policy

Raw expression data both bulk (gut biopsies) and single cell from purified CD4+ T-cells and plasma cells are deposited in the Gene Expression Omnibus (GEO; accession number GSE230524 for gut biopsy RNAseq and accession number GSE230569 for CD4 T-cell and plasma cell single cell gene expression sequencing and repertoire sequencing). Process flow cytometry, ELISpot, and clinical metadata are allocated in a Zenodo repository (DOI: 10.5281/zenodo.7857026). Individual-level data is available in these repositories without time limitation. GRCh38 can be found at https://www.ncbi.nlm.nih.gov/assembly/GCF_000001405.26/. GRCh37/hg19 can be found at https://www.ncbi.nlm.nih.gov/assembly/GCF_000001405.13/.

Research involving human participants, their data, or biological material

Policy information about studies with human participants or human data. See also policy information about sex, gender (identity/presentation), and sexual orientation and race, ethnicity and racism.

Reporting on sex and gender	Sex of each participant was self-reported, and we took careful consideration to ensure that there was a balance of sexes across diagnosis groups. Sex was used as a co-variate in the primary tissue transcriptional analysis which is the basis of all subsequent analysis. No sex-stratified analysis was performed as the proportion of patients identifying as female were included when comparing PSC to IBD (35% vs 38% without dysplasia, and 50% vs 57% with dysplasia) This sex distribution is consistent with the known sex distribution within PSC (~60% male). Disaggregate data on the sex of each participant is available in the metadata file publicly available on Zenodo.
Reporting on race, ethnicity, or other socially relevant groupings	Race and ethnicity were both self-reported in our study and included as co-variates in tissue transcriptional analysis which is the basis of all subsequent analyses. There was no significant difference in the distribution of races across patient groups (Extended Data Tables 1 and 2).
Population characteristics	Age, sex, race, and ethnicity of each patient was collected, and is summarized in Extended Data Tables 1 and 2. Individual-level data is available in the clinical and demographic metadata table avaiable on Zenodo.
Recruitment	Adults scheduled for a standard of care colonoscopy at UChicago Medicine (UCM) were screened for diagnosis and eligibility criteria for enrollment on a weekly basis. Exclusion criteria included: patients with active or chronic infections such as human immunodeficiency virus (HIV), hepatitis B (HBV), hepatitis C (HCV), or active, untreated Clostridia dificile; active infection with severe acute respiratory syndrome coronavirus 2 (SARS-CoV-2); intravenous or illicit drug use such as cocaine, heroin, or non-prescription methamphetamines; active use of blood thinners; severe comorbid diseases; patients on active cancer treatment; and patients who are pregnant. Approaching prospective patients was at the discretion of their treating physician and was not done in cases that would put patients at any increased risk, regardless of reason. Patients were approached the day of their procedure and informed, written consent was obtained prior to the procedure. No financial compensation was provided to participants.
Ethics oversight	Enrollment of patients at UChicago Medicine, collection of samples, and sample analysis were approved by the University of Chicago Institutional Review Board (IRB) and performed under IRB protocols 15573A and 13-1080. Samples collected at the Washington University School of Medicine were collected under the IRB 201111078. Samples collected at the Ichan School of Medicine at Mount Sinai were collected under GCO 14-0727.

Note that full information on the approval of the study protocol must also be provided in the manuscript.

Field-specific reporting

Please select the one below that is the best fit for your research. If you are not sure, read the appropriate sections before making your selection.

☒ Life sciences ☐ Behavioural & social sciences ☐ Ecological, evolutionary & environmental sciences

For a reference copy of the document with all sections, see nature.com/documents/nr-reporting-summary-flat.pdf

Life sciences study design

All studies must disclose on these points even when the disclosure is negative.

Sample size	No statistical method was used to predetermine sample size due to the rare nature of PSC.
Data exclusions	Samples from patients with an unclear diagnosis were retrospectively excluded from the study. If the same patient was sampled at multiple visits, only the first sample was used in the analysis of the tissue RNAseq. For the subsequent analyses, if the same patient was sampled on multiple visits, only a single sample was included per transcriptional cluster. Samples that did not pass quality control for transcriptional analysis were excluded.

Replication

Each sample acquired is from an individual at a specific time point, and is by nature irreproducible.

Randomization

The experiments were not randomized. Each subject was classified by diagnosis as well as transcriptional identity determined after processing of the samples but prior to the final analysis. Assignment to transcriptional cluster was unbaised (see methods).

Blinding

The investigators were not blinded to allocation during experiments and outcome assessment.

Reporting for specific materials, systems and methods

We require information from authors about some types of materials, experimental systems and methods used in many studies. Here, indicate whether each material, system or method listed is relevant to your study. If you are not sure if a list item applies to your research, read the appropriate section before selecting a response.

Materials & experimental systems

n/a	Involved in the study
☐	☒ Antibodies
☒	☐ Eukaryotic cell lines
☒	☐ Palaeontology and archaeology
☒	☐ Animals and other organisms
☒	☐ Clinical data
☒	☐ Dual use research of concern
☒	☐ Plants

Methods

n/a	Involved in the study
☒	☐ ChIP-seq
☐	☒ Flow cytometry
☒	☐ MRI-based neuroimaging

Antibodies

Antibodies used

The following directly conjugated antibodies were used to identify cell surface markers (clone, manufacturer, and catalog number, in parenthesis): mouse anti-human CD45-BV711 at 1:500 dilution (HI30, BD Biosciences 564357), mouse anti-human CD3-PE-Cy7 at 1:100 dilution (UCHT1, BioLegend 300420), mouse anti-human TCR α/β-BV421 at 1:20 dilution (IP26, BioLegend 306722), mouse anti-human CD4-BV510 at 1:50 dilution (SK3, BD Biosciences 562970), mouse anti-human CD8-BUV496 at 1:50 dilution (RPA-T8, BD Biosciences 612942), mouse anti-human CD19-PE at 1:50 dilution (HIB19, BD Biosciences 561741), mouse anti-human CD27-BV605 at 1:50 dilution (O323, BioLegend 302830), and mouse anti-human CD38-PerCP-Cy5.5 at 1:100 dilution (HIT2, BioLegend 303522). The following directly conjugated antibodies were used to identify intracellular markers (clone, manufacturer, and catalog number in parenthesis): mouse anti-human CD45-BV711 at 1:500 dilution (HI30, BD Biosciences 564357), mouse anti-human TCR α/β-BV421 at 1:20 dilution (IP26, BioLegend 306722), mouse anti-human CD4-BV510 at 1:50 dilution (SK3, BD Biosciences 562970), mouse anti-human CD8-BUV496 at 1:50 dilution (RPA-T8, BD Biosciences 612942), mouse anti-human IFNγ-PE at 1:100 dilution (4S.B3, eBioscience, supplied by ThermoFisher 12-7319-82), mouse anti-human TNFα-FITC at 1:100 dilution (Mab11, BioLegend 502906), mouse anti-human IL-17A-APC at 1:50 dilution (BL168, BioLegend 512334), and rat anti-human Foxp3-PE-Cy7 at 1:20 dilution (PCH101, eBioscience, supplied by ThermoFisher 25-4776-42). Antibodies used in ELISpot are the following: polyclonal goat-anti human IgA, IgG, and IgM antibodies (KPL, supplied by SeraCare 5210-0160) and Biotin-conjugated polyclonal goat anti-human IgA, IgG, or IgM (Southern Biotech, 2050-08, 2040-08, and 2020-08 respectively).

Validation

The available flow cytometry plots and paper citations using the antibody clone were reviewed prior to purchase to determine suitability for experimentation. Only antibodies demonstrating clear separation between positive and negative populations were used. Prior to experimentation, each antibody was serially diluted and used to stain PBMCs from whole blood (with PMA-ionomycin stimulation for cytokines) to determine the optimal dilution and to confirm that the proportion of stained cells was consistent with what was anticipated based on published data.

Flow Cytometry

Plots

Confirm that:

☒ The axis labels state the marker and fluorochrome used (e.g. CD4-FITC).

☒ The axis scales are clearly visible. Include numbers along axes only for bottom left plot of group (a 'group' is an analysis of identical markers).

☒ All plots are contour plots with outliers or pseudocolor plots.

☒ A numerical value for number of cells or percentage (with statistics) is provided.

Methodology

Sample preparation

Colonic lymphocytes were isolated via mechanical disruption and enzymatic digestion. Briefly, colonic biopsies were twice shaken at 250 revolutions per minute (rpm) for 30 minutes at 37C in 7mL RPMI 1640 (Fisher Scientific) supplemented with 1% dialyzed fetal bovine serum (Biowest), 2mM EDTA (Corning), and 1.5 mM MgCl2 (Thermo Fisher Scientific). This fraction was discarded. Subsequently, the tissue was digested in two sequential shakes at 250rpm at 37C for 30 minutes in 15mL

	RPMI 1640 supplemented with 20% fetal bovine serum and 1mg/mL collagenase type IV, from Clostridium histolyticum (Sigma-Aldrich). After each digestion, the solution was filtered, centrifuged, and then combined for downstream experimentation. This fraction was considered the lamina propria fraction.
Instrument	BD FACSAria Fusion, Cytek Aurora, or BD LSRFortessa
Software	All flow cytometry data were analyzed using FlowJo software version 10.7.2 (Tree Star).
Cell population abundance	The post-sort fraction of CD4 amongst all CD45+ live singlets was on average 20.1% with a range from 10.47 to 29.2%. The post-sort fraction of plasma cells amongst all CD45+ live singlets was on average 34.82% with a range from 18.28 to 57.76%. Sample purity was determined by running a small fraction of the sorted cells back on the flow cytometer. Purity was considered acceptable if above 95%. The purity of the samples was further confirmed during the analysis of the single cell RNA sequencing, as the transcriptional profiles of the sorted cells were analyzed for coherence with the anticipated transcriptome of a CD4 T-cell or a plasma cell respectively.
Gating strategy	CD4 T-cells were CD45+ LIVE/DEADnegative > FSC vs SSC > singlets > CD3+ CD19negative > CD4+ CD8negative and plasma cells were CD45+ LIVE/DEADnegative > FSC vs SSC > singlets > CD3negative > CD38+ CD27+.

☒ Tick this box to confirm that a figure exemplifying the gating strategy is provided in the Supplementary Information.
